# mRNA-based therapeutics: powerful and versatile tools to combat diseases

**DOI:** 10.1038/s41392-022-01007-w

**Published:** 2022-05-21

**Authors:** Shugang Qin, Xiaoshan Tang, Yuting Chen, Kepan Chen, Na Fan, Wen Xiao, Qian Zheng, Guohong Li, Yuqing Teng, Min Wu, Xiangrong Song

**Affiliations:** 1 Department of Critical Care Medicine, Frontiers Science Center for Disease-related Molecular Network, State Key Laboratory of Biotherapy and Cancer Center, West China Hospital, Sichuan University, Chengdu, China; 2 Department of Biomedical Sciences, School of Medicine and Health Sciences, University of North Dakota, Grand Forks, ND 58203 USA

**Keywords:** Molecular biology, Cell biology

## Abstract

The therapeutic use of messenger RNA (mRNA) has fueled great hope to combat a wide range of incurable diseases. Recent rapid advances in biotechnology and molecular medicine have enabled the production of almost any functional protein/peptide in the human body by introducing mRNA as a vaccine or therapeutic agent. This represents a rising precision medicine field with great promise for preventing and treating many intractable or genetic diseases. In addition, in vitro transcribed mRNA has achieved programmed production, which is more effective, faster in design and production, as well as more flexible and cost-effective than conventional approaches that may offer. Based on these extraordinary advantages, mRNA vaccines have the characteristics of the swiftest response to large-scale outbreaks of infectious diseases, such as the currently devastating pandemic COVID-19. It has always been the scientists’ desire to improve the stability, immunogenicity, translation efficiency, and delivery system to achieve efficient and safe delivery of mRNA. Excitingly, these scientific dreams have gradually been realized with the rapid, amazing achievements of molecular biology, RNA technology, vaccinology, and nanotechnology. In this review, we comprehensively describe mRNA-based therapeutics, including their principles, manufacture, application, effects, and shortcomings. We also highlight the importance of mRNA optimization and delivery systems in successful mRNA therapeutics and discuss the key challenges and opportunities in developing these tools into powerful and versatile tools to combat many genetic, infectious, cancer, and other refractory diseases.

## Introduction

Messenger RNA (mRNA) is a type of single-stranded ribonucleic acid that is transcribed from a strand of DNA, which carries the coding information for protein synthesis to be further transcribed and processed into functional proteins.^1^ In vitro transcription (IVT) mRNA was successfully transcribed and expressed in mouse skeletal muscle cells, which establishes the feasibility of mRNA therapy.^2^ mRNA-based therapeutics were proposed when mRNA could be successfully transfected and produce an immune response in a dose-dependent manner by direct injection into mice to express therapeutic proteins.^3^ An mRNA-based approach can theoretically produce any protein/peptide *via* the protein synthesis machine processed in the transfected cell in vitro or in vivo.^4^ Unlike DNA-based drugs, mRNA transcripts have a relatively high transfection efficiency and low toxicity because they do not need to enter the nucleus to be functional.^5^ Importantly, mRNA has no potential risk of accidental infection or opportunistic insertional mutagenesis.^6^ In addition, mRNA has broad potential for treating diseases requiring protein expression and higher therapeutic efficacy due to its continuous translation into encoded proteins/peptides to trigger long-lasting expression compared to transient traditional protein/peptide drugs.^7^ Apparently, these advantages of mRNA over DNA or protein/peptide enable the rapid entry of mRNA-based technology and products into various branches of the biomedical fields, which will benefit all aspects of human life, especially millions of patients suffering from incurable diseases.

**Fig. 1:**
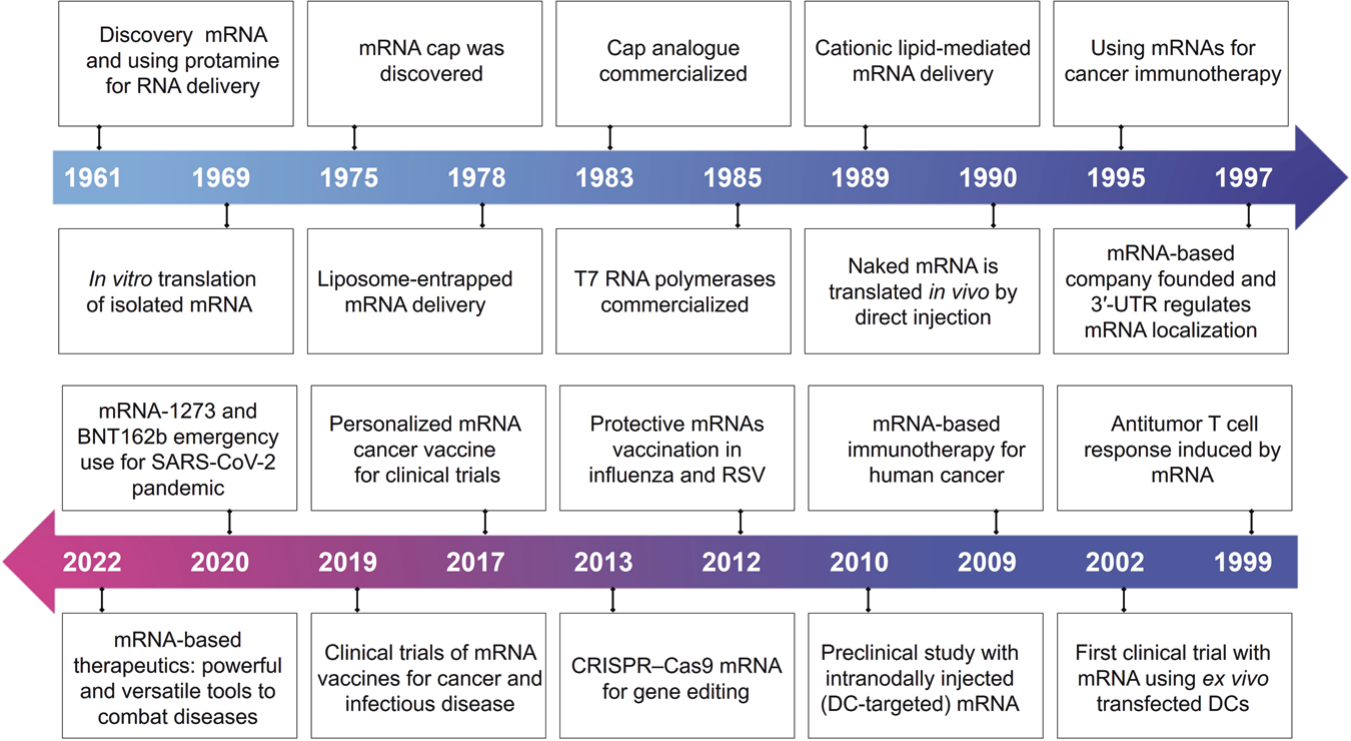
Key discoveries and advances in mRNA-based therapeutics. The development of mRNA-based therapeutics can be divided into three stages. Phase 1, mRNA discovery, in vitro synthesis and nucleic acid delivery system construction (1961–1990), including discovery mRNA^523^ and using protamine for RNA delivery,^524^ in vitro translation of isolated mRNA,^525^ mRNA cap was discovered,^526^ Liposome-entrapped mRNA delivery,^527^ Cap analog commercialized, T7 RNA polymerases commercialized, Cationic lipid-mediated mRNA delivery,^528^ Naked mRNA is translated in vivo by direct injection.^529^ Phase2 (1990-2019), accumulated knowledge with the continuous attempts and diverse applications, especially protein replacement therapies and vaccination approaches for cancer and infectious diseases, including using mRNAs for cancer immunotherapy,^5^ mRNA-based company founded and 3′-UTR regulates mRNA localization,^530^ antitumor T cell response induced by mRNA,^531^ first clinical trial with mRNA using ex vivo transfected DCs,^532^ mRNA-based immunotherapy for human cancer,^533^ preclinical study with intranodally injected DC-targeted mRNA,^534^ protective mRNAs vaccination in influenza^240^ and respiratory syncytial virus,^98^ CRISPR–Cas9 mRNA for gene editing,^535^ personalized mRNA cancer vaccine for clinical trials.^330^ Phase 3, mRNA-based therapeutics, as a disruptive therapeutic technology, is becoming powerful and versatile tools for therapy diseases (2019 to present), including clinical trials of mRNA vaccines for cancer and infectious disease, mRNA-1273,^536^ and BNT162b emergency use for SARS-CoV-2 pandemic^537^

Nevertheless, insufficient knowledge of mRNA structure instability and immunogenicity has dampened some of the promises and impeded the pace of mRNA-based therapeutics to combat diseases.^8^ mRNA is a negatively charged macromolecule that is susceptible to ubiquitous RNases. Hence, it is quite difficult for mRNA to pass through the anionic cell membrane and translate functional proteins in the cytoplasm (<1/10,000 mRNAs of the initial input).^9^ In addition, mRNA can also induce an immune response with associated toxicity, which greatly restricts the development of mRNA-based therapeutics.^10^ Engineering precision carriers for mRNA-based drug delivery reveal a critical role in improving immunogenicity and instability and overcoming cellular barriers.^11^ Recently, based on the important role of mRNA vaccines in controlling the severe acute respiratory syndrome coronavirus 2 (SARS-CoV-2) pandemic, humans benefited from a large number of mRNA vaccines for infectious diseases on structural and chemical modifications, which have also greatly fueled enthusiastic efforts in the development of mRNA-based therapeutics to improve their stability, translation efficiency and immune response^12^ (Fig. 1). Simultaneously, mRNA can be successfully delivered into a variety of cells with continuous breakthroughs of delivery carriers.^13^ Numerous technologies have also been developed to improve mRNA therapeutic efficacy and the instability of mRNAs. Hence, it is necessary to draw a comprehensive landscape of the current status and analyze the general design approaches of mRNA-based drugs.

Our lab has been committed to promoting mRNA-based therapeutics to become powerful and versatile tools to combat diseases, especially in gene therapy and immunotherapy.^14^ We have developed diverse novel targeted delivery nanoparticles^15^ and constructed receptor-binding domain (RBD)-encoding mRNA formulated in liposomes to prevent and treat the SARS-CoV-2 pandemic.^16^ In this review, we comprehensively summarize the recent progress towards mRNA design and synthesis, as well as the enabling of mRNA delivery technologies. Likewise, we point out the key issues and challenges facing the future of the platform, including mRNA optimization and application in specific diseases and populations, offering novel insight into the design, test, and development of mRNA therapeutics.

## mRNA design and manufacture

**Fig. 2:**
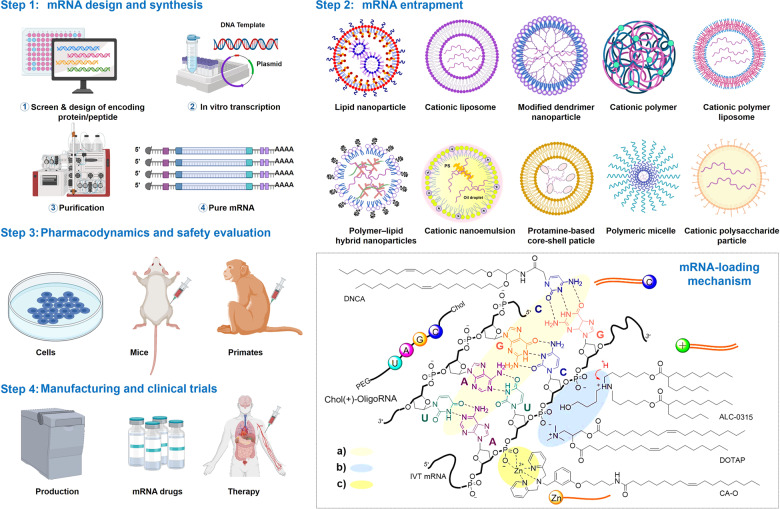
mRNA drugs production pipeline. The encoding of peptide/protein is designed and inserted into a plasmid DNA construct. Plasmid DNA is transcribed into mRNA by bacteriophage polymerases in vitro, and mRNA transcripts are purified by high-performance liquid chromatography (HPLC) or nanoprecipitation to remove contaminants and reactants. Subsequently, purified mRNA is entrapped in various vehicles. The interactions between vehicles and mRNA can be divided into three types: (a) electrostatic adsorption with phosphate ions of the ribonucleotides; (b) complementary paired hydrogen bonding with bases of the ribonucleotides; and (c) coordination with the phosphate ions. Thus, vehicles for mRNA delivery consist of the following categories: cationic compounds, such as cationic lipids, ionizable lipids, and cationic polymers. Nucleoside-based lipids, e.g., DNCA, or nucleoside-based amphiphilic polymers, e.g., Chol(+)-oligoRNA. Metal-based compounds provide vacant orbitals to coordinate with phosphate ions. Furthermore, the efficacy, pharmacology, and safety of mRNA drugs were evaluated in vaccinated mice and primates. Finally, the scale-up manufacturing of mRNA therapeutics is conducted and followed by clinical trials^262^

The development of mRNA-based therapeutics mainly includes mRNA design, synthesis, mRNA entrapment, pharmacodynamics, pharmacokinetics, safety evaluation in vivo and in vitro, manufacturing, and clinical trials (Fig. 2). mRNA design and synthesis are crucial steps in mRNA-based medicines. mRNA features five functional regions, including the 5′ cap, the 3′ poly(A) tail, the open reading frame (ORF) flanking, and 3′ untranslated regions (UTRs), whose elements mediate the translation efficacy and decay rate of mRNA.^6^ Notably, obtaining highly biologically active RNA usually depends on reliable design and preparation.^17^ In this section, we focus on recent advances and discuss the challenges of mRNA design and preparation. In addition, nucleoside modification and purification are also reviewed (Table 1), which are widely applied to adjust the different demands for mRNA immune-stimulation in various therapies.

**Table 1:** Critical quality controls in the preparation of mRNA drugs

Composition	Quality control items	Outcome
Antigen-encoded mRNA	Codon optimization	Translation efficiency
	Nucleic acid quantity	Translation efficiency
	pH at mRNA synthesis stage	Translation efficiency
	mRNA sequence identity	Translation efficiency
	mRNA sequence integrity	Translation efficiency
	Poly A tail length	Translation efficiency
	Efficiency of 5′ caping	Translation efficiency
	5′-UTRs and 3′-UTRs optimization	Translation efficiency
	mRNA purity	Translation efficiency
	Residual DNA template	Translation efficiency
Lipid delivery system	Mass spectrometry analysis	Transfection efficiency
	Nuclear magnetic resonance analysis	Transfection efficiency
	Lipid component identities	Transfection efficiency
	Lipid electric charge	Targeting
	Lipid ratios	Targeting
	Isoelectric point	Stability
	Micromorphology	Uniformity
	Lipid impurities	Transfection efficiency
	Distribution	Targeting
	Transfection efficiency in vivo	Transfection efficiency
	Transfection efficiency in vitro	Transfection efficiency
mRNA-lipid nanoparticle drugs	Encapsulation efficiency	Loading capacity
	Particle size	Uniformity
	Zeta potential	Stability
	Storage conditions	Clinical application
	Release principle	Therapeutic potential

### The structural elements of mRNA

**Fig. 3:**
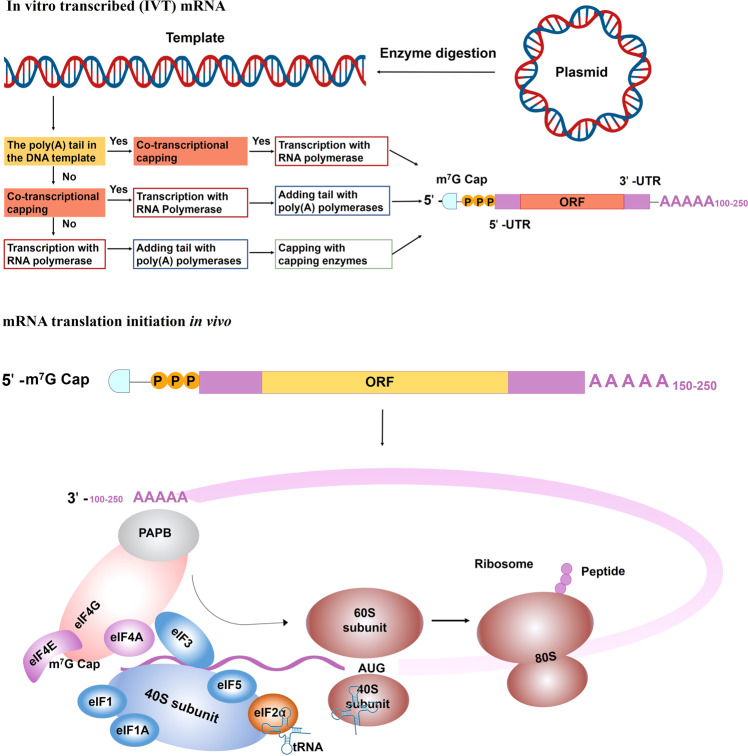
In vitro transcribed (IVT) mRNA and translation initiation.IVT mRNA preparation includes several steps, plasmid cloning, plasmid linearization, in vitro transcription, 5′ capping, and the poly(A) tail adding. Transcription, capping and the tail adding can combine into one, two or three steps that depend on the design of synthesis routes.^2^ After entering into the cell, mRNA translation can be initiated in an eIF4F-dependent manner to recruit a preinitiation complex (PIC). The 43S PIC is formed by 40S ribosomal subunit, the eukaryotic translation initiation factors (eIF, including eIF1, eIF1A, eIF3, eIF5) and the ternary complex, including a trimeric complex comprising eIF2 that contains α-, β-, and γ-subunits, initiating methionyl tRNA (tRNAiMet), and GTP. eIF4F is a complex composed of eIF4A, eIF4E and eIF4G. eIF4E binds to mRNA cap. eIF4G interacts with eIF3 and poly(A)-binding protein (PABP) that binds to the 3′ poly(A) tail. These interactions result in mRNA circularization and 48S PIC assembly. The 48S PIC ribosomal subunit scans and finds the start codon with the help of eIF4A helicase to resolve secondary mRNA structure in the 5′ UTR. Then, eIFs are released and 60S ribosomal subunit joins to initiate translation elongation by forming 80S ribosome^21^

mRNA is produced by the transcription process. The precursor mRNA is synthesized in eukaryotes when RNA polymerase converts genes into primary mRNA transcripts in vivo, which usually still contains noncoding sequence introns, and are further removed to become mature mRNA by mRNA processing, including 5′ mRNA capping, modifications, splicing, and A-to-I editing.^18^ IVT mature mRNA preparation includes several steps, linear DNA template obtainment, IVT, 5′ capping, and poly(A) tail adding. After the mRNA is transferred into the cell, poly(A)-binding protein (PABP) binds to the poly(A) tail and interacts with eukaryotic translation initiation factors (eIFs). The interaction of eIFs with the 5′ cap, UTRs, PABP, initiator methionyl transfer RNA (tRNA), and 40S ribosomal subunit, render mRNA circularization and the formation of an initiation complex. After 40S ribosomal subunit scans the transcription initiation codon, 60S ribosomal subunits are recruited and eIFs are released to start amino acid chain extension.^19^ Mature mRNA includes the coding region, UTR, the poly(A) tails, and the 5′ cap that can be recognized by ribosomes and carried by tRNA to create proteins. As in DNA, genetic information in mRNA is contained in the sequence of nucleotides that are arranged into codons consisting of three ribonucleotides each. Accordingly, IVT mRNA is performed to complete the transcription of RNA in vitro by stimulating the mechanism of eukaryotic mRNA synthesis to ensure the expression of mRNA in vivo (Fig. 3). Therefore, the optimization of mRNA is essential for successful mRNA-based therapeutics.

### mRNA translation and decay

**Fig. 4:**
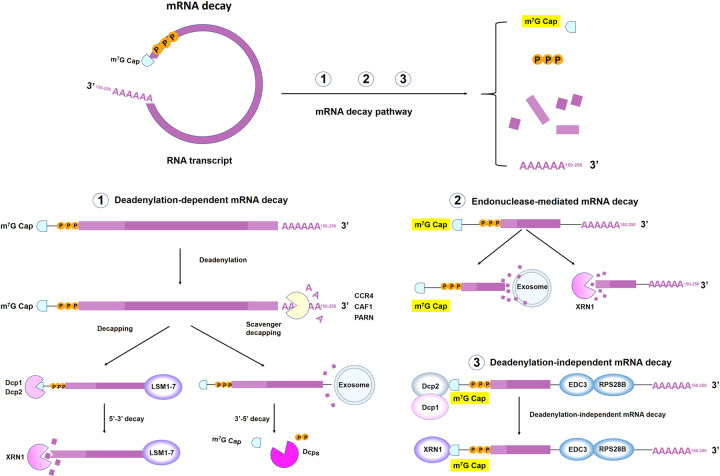
Mechanisms of mRNA decay. Degradation of messenger mRNA plays an essential role in regulating sustained mRNA expression. mRNA is generally degraded in the following three pathways: **①** Deadenylation-dependent mRNA decay: The poly(A) tail is removed by deadenylase activity (such as CCR4, CAF1 or PARN). The LSM1-7 complex associates with the 3′-end of the mRNA transcript to induce decapping by the Dcp1–Dcp2 complex and is then degraded by exoribonuclease XRN1. Alternatively, deadenylated mRNA can be degraded by exosomes. **②** Endonuclease-mediated mRNA decay: The mRNA is cleaved into two fragments, and then the fragments are degraded by XRN1 and exosomes.^538^ **③** Deadenylation-independent pathways require recruitment of the decapping machinery. RPS28B interacts with the enhancer of decapping-3 (Edc3) to engage the decapping enzyme. Subsequently, the mRNA is degraded by XRN1^538^

Eukaryotic mRNA translation initiation is an exquisitely regulated process involving the assembly of a multiprotein–RNA complex that directs ribosomes to the initiation codon.^20^ Generally, Cap-dependent translation begins with the cap recognition by eukaryotic initiation factor 4F (eIF4F) and the assembly of the preinitiation complex (PIC), which consists of the ternary complex, the 40S ribosomal subunit, eIF1, eIF1A, eIF3 and eIF5.^21^ eIF4F consists of eIF4A, eIF4E and eIF4G, which facilitates PIC recruitment by eIF4E–cap and eIF4G–eIF3 interactions. eIF4F renders mRNA circularization by interacting with the 5′ cap through eIF4E and the PABP that binds with the poly(A) tail.^22^ 40S ribosomal scans the 5′-UTR and recognizes initiation codon with the help of eIF4A to unwind the secondary structure of the 5′-UTR, subsequent, 40S ribosomal subunit scans the transcription initiation codon, 60S ribosomal subunits are recruited and eIFs are released to start amino acid chain extension.^23^ Then, mRNA is decoded in a ribosome to produce a specific amino acid chain or polypeptide. There is a balance between the processes of translation and mRNA decay^24,25^ (Fig. 4). It has previously been implicated that these structural elements that are being actively translated also intimately connect to mRNA decay, especially the 5′ cap and the poly(A) tail.^26^ The 5′ cap protects mRNA from 5′ to 3′ exoribonucleases,^27^ and the length of the poly(A) tail determines the 3′ to 5′ exonucleolytic decay.^28^ Based on the vital importance of these functional elements, numerous studies have focused on the optimization of mRNA structure, such as developing a series of 5′ cap analogs, changing the poly(A) tail length, screening feature UTRs and encoding various functional peptides or viral replication machinery in ORFs.^29^

### mRNA design

#### The 5′ cap

**Fig. 5:**
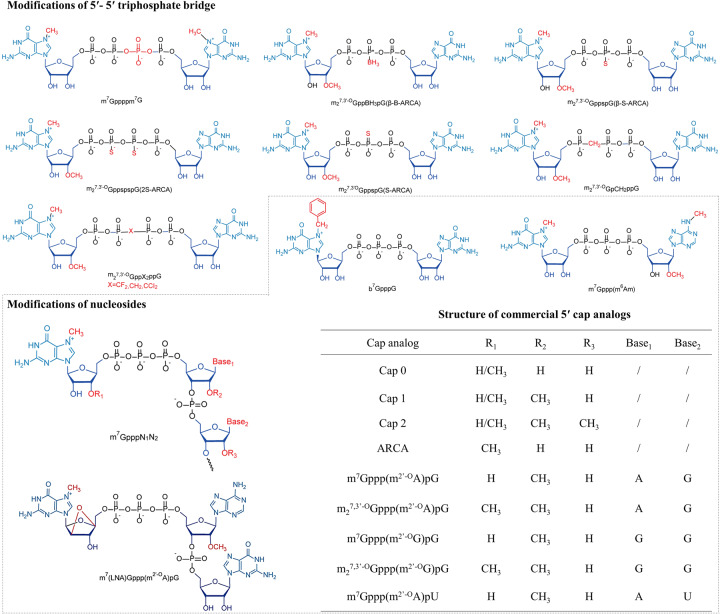
Commercialization and commonly used Cap. The 5′ cap of mRNA is critical to improve mRNA stability and promote translation efficiency. Modification of the 5′-5′ phosphate bridge can increase the resistance to DcpS and Dcp1/Dcp2, but the translation efficiency may not necessarily increase (such as the introduction of methylene groups on the phosphate bridge). The modification of ribose nucleosides also plays essential functions in mRNA translation by recruiting translation initiation factors, such as the methylation modification on the N7 position of the guanosine cap and the ribose-2′O position of the first nucleotide (Cap 1), increasing the affinity for eIF4E and thereby improving translation efficiency^116,539^

The 5′ caps are located at the 5′ terminus of mRNA with different degrees of methylation.^30^ 5′ caps (m^7^G ppp) contain a 7-methylguanosine (m^7^G) attaching the following nucleotide through a 5′-5′ triphosphate bridge (ppp) in eukaryotes^31,32^ (Fig. 5). The cap combines eIF4E via the hydrophobic cation–π interactions of m^7^G and the negative electrostatic charge of the triphosphate bridge during translation initiation.^33^ For cap removal, the triphosphate bridge is the major target mRNA decapping enzyme in eukaryotic cells. Dcp1/2 and DcpS: Dcp1/2 cleave α- and β-phosphate, and DcpS cleaves β- and γ-phosphates.^34,35^ Therefore, numerous strategies for mRNA structure optimization have been applied to optimize m^7^G or the triphosphate bridge to achieve cap analogs with high affinity for eIF4E and low susceptibility for decapping enzymes.^36^ Rydzik et al. increased the cap resistance to decapping by substituting the oxygen atom of triphosphates with dihalogenmethylenebisphosphonate.^37^ In addition, the modification of m^7^G is an essential approach to improve mRNA translation. It has previously been reported that the translation efficiency is significantly enhanced by replacing the 7-methylated guanosine (m^7^G) with 7-benzylated guanosine^38^ and further increased by 2-fold by attaching the m^7^G with another m^7^G *via* tetraphosphate (m^7^Gppppm^7^G), whose analogs have a higher affinity for eIF4E compared to natural eukaryotic 5′ caps.^39^ The bridged oxygen atoms between α-β or β-γ phosphate were, respectively, replaced with methylene to give rise to m^7^GpCH_2_ppG or m^7^GppCH_2_pG to prevent mRNA from Dcp1/2 or DcpS degradation.^40^ Dithiodiphosphate modification are also introduced to the tri- or tetraphosphate bridge, which decreased cap sensitivity to Dcp1/2, and improved mRNA translation.^36^ In addition, phosphorothioate cap analogs increase the stability and translational efficiency of RNA vaccines in immature dendritic cells (DCs).^41^ Notably, phosphorothioate substitution is position sensitive, which is possibly associated with stereochemistry in catalysis.^36^

#### The poly(A) tail

Poly(A) tails generally consist of 10–250 adenine ribonucleotides. Poly(A) tails are dynamic additions to mRNA that their length plays a crucial role in regulating mRNA translation efficacy and protein expression.^42,43^ Mechanically, the 3′ -end poly(A) tail combines with PABPs and subsequently interacts with the 5′ cap through the translation initiation factors eIF4G and eIF4E, which promotes a “closed-loop structure” and regulates the translation efficiency of mRNA.^44^ Mockey et al. are the first to observe a positive correlation between the length of poly(A) tails and translation efficacy by adding a poly(A) tail of 100 instead of 64 adenosines in cis, increasing the protein level by approximately 35-fold.^45^ Similarly, the poly(A) of 120 units is more conducive to the formation of stable and efficient translation mRNA compared to the 51 nt and 42 nt tails,^46^ and the 325-nucleotide poly(A) tail shows higher efficacy than the 172-nucleotide tail.^47^ Interestingly, the length of poly(A) is not always positively correlated with mRNA instability and attenuation. Traditionally, it was considered necessary for poly(A) tails to contain at least 20 nt to achieve sufficient mRNA translation, but the poly(A) tails of stabilizing β-actin are less than 20 nucleotides, and the poly(A) tails of 425 nt and 525 nt merely contribute to transfection efficiency than 120 nt poly(A) tails in human primary T cells.^47–49^

#### 5′-UTRs and 3′-UTRs

The UTRs at the 3′ and 5′ terminals of mRNAs do not directly encode proteins but play important roles in regulating mRNA translation and protein expression.^50^ UTRs participate in the subcellular localization of mRNA, and regulating translation efficiency and mRNA stability.^51^ Both the 5′-UTR and 3′-UTR regulate protein expression levels, and the 5′-UTR is primarily involved in initiating the translation process,^52^ while the 3′-UTR mostly affects the stability and half-life of the mRNA.^53^ The 5′ cap triggers ribosome binding and subsequently recognizes the initiation sequence for protein synthesis during translation. Furthermore, the internal ribosome entry site in the 5′-UTR can also recruit ribosomes and initiate translation in a cap- and eIF4E-independent manner.^54^ The strongest Kozak sequence is widely used to improve mRNA translation. Foroughmand et al. improved protein expression by replacing the Kozak sequence of the human beta-globin 5′-UTR with the strongest sequence.^55^ A library of 10 UTR variants was constructed to validate the effect of UTR on the expression of therapeutic mRNA, and found that 5'UTRs containing the complement factor 3 (C3) and cytochrome p4502E1 significantly increased protein translation regardless of 3'UTR modifications.^56^ Similarly, optimization of the 3′-UTR can also enhance mRNA stability and translation duration. The stability of the mRNA is enhanced due to the discontinuous pyrimidine-rich sequence in the 3′-UTR of α-globin, and the β-globin in mRNA contributes to the increased duration of protein expression.^57,58^ More efficacious strategies are developed for increasing protein production and mRNA stability by adding two consecutive β-globin 3′-UTRs arranged head-to-tail to mRNA compared to one β-globin 3′-UTR. Notably, the improvement is cell-type dependent, which significantly increases protein expression in mature DCs but slightly immature DCs.^46^ Conversely, eGFP mRNA with two repeated β-globin 3′-UTRs produces less protein than mRNA with β-globin 5′-UTRs in human pluripotent stem cells (PSCs).^59^ However, two repeated *cytochrome b-245 alpha polypeptide* (CYBA) 3′-UTRs had lower protein production in A549 cells, compared to the single 3′-UTR.^60^ Moreover, the 5′-UTR and 3′-UTR influence each other on protein expression.^56^ Taken together, the 5′-UTR contributes to the regulation of protein expression depending on the systems and cell types.

Trepotec et al. designed a series of short 5′-UTRs by inserting or altering less than two ribonucleosides based on the Kozak sequence. Two short 5′-UTRs were either better or equally effective than the human *alpha globin* 5′-UTR.^61^ Ferizi et al. evaluated UTRs from five natural long-lived mRNAs and found that the UTRs from human CYBA have the highest and most stable protein expression in NIH3T3 cells and A549 cells.^60^ Schrom et al. compared the effectiveness of a minimal 5′-UTR, a human *alpha globin* 5′-UTR and CYBA 5′-UTR, which resulted in higher protein expression by optimizing coding.^62^ Segovia et al. tried to reduce the immune stimulation of mRNA using the 5′-UTR from the Venezuelan equine encephalitis (VEE) virus.^63^ Asrani et al. used plasmids and IVT mRNA to screen effective UTRs, while they found different protein expressions driven by plasmids and IVT mRNA in HepG2 cells.^56^ Notably, researchers tried to design effective UTRs with the help of bioinformation and machine learning.^64,65^

#### The open reading frame

The design of the ORF has largely focused on codon optimization and the introduction of functional peptides as well as replication processes.^66^ Codon optimization is an extensively used but controversial approach for translation improvement.^67^ mRNA translation efficiency was improved by replacing rare codons with synonymous codons decoded by tRNA with higher abundance in ORF,^68,69^ but it may change protein conformation and give rise to novel peptides with unknown biological activity in vivo.^68,70^ Increasing the GC content by replacing rare codons in ORFs protects mRNA from endoribonuclease degradation and enhances mRNA protein expression in vivo.^71,72^ In addition, functional peptides are crucial for mRNA drugs and the signal peptides encoded by mRNAs are necessary for proteins that exert functions outside of the cells.^73^ Accordingly, optimization of mRNA for improving the function of therapeutic mRNA by introducing signal peptides to ORF regions is required. Trafficking signal peptides and protein segments are also widely applied for the improvement of antigen presentation in mRNA vaccines.^74^ Kreiter et al. improved the trafficking property of protein antigens by encoding a secretion signal and the transmembrane cytoplasmic domain of the MHC I molecule in the ORF, which increased antigen presentation by ~10-fold in DCs and improved the antitumor efficacy of mRNA vaccines in mice.^75^ Other functional peptides are also used to enhance cytoplasmic expression: the β2-microglobulin of MHC I molecules and the signal peptide of DC lysosomal-associated membrane protein.^76^ Together, the quality control of mRNA at each step is directly related to its efficacy; therefore, mRNA production and preparation is the key to mRNA-based therapeutics.

#### RNA chemical formula design

##### Self-amplifying RNA

Compared to conventional mRNA, self-amplifying RNA (saRNA) is another kind of mRNA molecule with a different structure.^77,78^ saRNA primarily originates from alphavirus structures and is constructed by replacing the gene sequence coding for virus structural proteins with the gene sequence of interest.^79^ Alphaviruses are positive-sense, single-stranded RNA viruses with self-amplifying ability, which is performed by a sequence of nucleotides coding for nonstructural proteins (nsP1-4).^80^ These nonstructural polyproteins function as replicases and replicate virus structural proteins through RNA-dependent RNA synthesis.^81^ Therefore, saRNA can produce a large amount of protein of interest in an effective way by using the innate nature of alphaviruses.

The basic elements of saRNA are the 5′ cap, 5′-UTR, sequence coding for nsP1-4, subgenomic promoter sequence, ORF with GOI, 3′-UTR, and 3′ poly(A) tail.^82^ The major difference between saRNA and conventional mRNA is the replicase sequence. The functions of individual nsP1-4 have been partially revealed: nsP1 plays a role in capping, nsP2 gains helicase activity, nsP3 is essential in the assembly of the replication complex and may interact with other proteins to prevent host cell-inhibiting pressure, and nsP4 obtains RNA-dependent RNA polymerase activity.^83–87^ All of the nonstructural proteins play an essential role in the function of saRNA. After saRNA is transfected into the cell, the sequence of nsP1-4 is translated into the nsP1-4 polyprotein, which functions as the precursor of the replicase complex, and subsequently, the nsP1-4 polyprotein is cleaved by nsP2, producing the nsP1-3 polyprotein and nsP4.^85^ This generated early phase replicase complex transcribes the original positive-sensed RNA strand into a negative-sensed RNA strand, and the latter strand is then used as the template for subsequent replication.^88,89^ After the nsP1-3 polyprotein is further cleaved into individual nsP1, nsP2, nsP3, together with nsP4, they form the cleaved replicase predominantly involved in the production of positive-strand synthesis.^90,91^

The greatest advantage of saRNA is the “dose-sparing” effect. Researchers in Imperial College London formulated the saRNA coding for S protein in the lipid nanoparticle (LNP) as vaccines against SARS-CoV-2, showing high efficiency in inducing neutralizing antibody titers.^92^ The same effect has also been shown in mRNA vaccines against ZIKV^93^ and influenza.^94^ However, the main challenge for saRNA is its longer sequence (usually 9–12 kb) compared to conventional mRNA. Some researchers have made some efforts to address this issue. Beissert et al. developed a novel bipartite vector system using trans-amplifying RNA.^95^ The vector system splits into two strands; one codes for the replicase with its enzyme activity provided by the second strand, and the other codes for the GOI that will be transamplified by the first strand.^96^ This work on saRNA structure showed the same efficacy as the single vector system while providing an easy, time- and cost-efficient manufacturing process. Li et al. optimized the replicon by identifying six mutations in nonstructural proteins of the VEE replicon that promoted subgenome expression in cells.^97^ Overall, saRNA is an attractive tool for transient expression of the target protein, generating stable cell lines expressing heterologous proteins from continuously replicating RNA, and developing recombinant vaccines.^79,98^ For example, Li et al. used saRNA to code the light and heavy chains of neutralizing anti-SARS-CoV-2 CB6 antibody simultaneously under the control of two identical subgenomic promoters.^99^ Together, saRNA has great absolute advantages in the continuous expression of proteins and long-lasting efficacy compared with other RNA chemical formula design, but the large nucleic acid sequence limits its application. Therefore, it still remains challenging for this promising technology.

##### Circular RNA, noncoding RNAs, and competitive endogenous RNA

Circular RNAs (circRNAs) are single-stranded, covalently closed RNA molecules that are ubiquitous in species ranging from viruses to mammals. CircRNAs, act as protein decoys, scaffolds and recruiters, exert biological functions by acting as transcriptional regulators, microRNA sponges, and protein templates. CircRNA is generated by back-splicing, in which the 3′-end of an exon ligates to the 5′-end of its own or an upstream exon through a 3′,5′-phosphodiester bond, forming a closed structure.^100^ The unique structure of circRNAs gives them greater stability, longer half-life, and greater RNase R resistance, which are linear mRNAs deficient and desired.^101^ Noncoding RNA (ncRNA) is an RNA molecule that is not translated into a protein, but affects normal gene expression and disease progression, including microRNA, intronic RNA, repetitive RNA, and long ncRNA.^102^ LncRNAs function as competing endogenous RNAs (ceRNAs) by competitively occupying shared binding sequences for miRNAs.^103^ CircRNA Cdr1as functions as a competitive endogenous RNA to promote hepatocellular carcinoma (HCC) progression.^104^ Research has shown that the complicated circRNA-miRNA-mRNA network revealed an important role in regulating Hantaan virus infection.^105^ circRNA-lncRNA-miRNA-mRNA ceRNA regulatory network was identified as novel prognostic markers for acute myeloid leukemia (AML).^106^ Currently, ncRNA-based therapeutics mainly regulates the expression of key proteins to treat diseases. The therapeutic potential of ncRNA has been recognized for more than forty years, few drugs have received approval due to high off-target effects.^107^ Although there is no report on the combination therapy strategy of mRNA and circRNA or ncRNAs. It may be an important means to achieve precise and individualized treatment by co-delivering them to form a regulatory network or complex, which is worthy of further exploration.

### mRNA manufacture

#### mRNA synthesis and optimization

IVT mRNA is performed with linearizing plasmid DNA templates or PCR templates requiring at least a promoter and the corresponding mRNA construct sequence.^2,108^ IVT mRNA is carried out by adding polymerases (T7, T3, or SP6) but requires additional capping.^108^ Uncapped mRNA is rapidly degraded by RNase and contains a 5′-ppp group, which causes greater immune stimulation and can be treated with phosphatase to reduce undesirable efficacy.^109,110^ Two methods are implemented for the capping of IVT mRNA: co-transcriptional capping and posttranscriptional capping.^111,112^ Cap dinucleotide mixtures containing four other nucleoside triphosphates (NTPs) are incorporated at the 5′ end of the RNA with RNA polymerase during co-transcriptional capping.^113^ A label-free method was described to identify the 5′-end cap and the orientation of mRNA.^114^ Co-transcriptional capping processing has permitted coordinated transcription with mRNA capping, but its disadvantages are the competitive incorporation of GTP nucleosides, which impairs capping efficiency.^111^

Intriguingly, GTP first binds to RNA chains *via* a 5′-5′ triphosphate bond and then 7-methylation of the 5′ terminal guanosine in posttranscriptional capping.^115^ Capping enzymes from vaccinia virus are widely used to cap mRNA, have high end-capping efficiency and are able to completely cap mRNA with cap-0.^116^ Furthermore, it is necessary to consider mRNA immune stimulation, and cap-specific 2′-O methyltransferase is used to produce cap 1 or cap 2 based on cap 0, which reduces mRNA immunogenicity.^117,118^ The polymerase initiates transcription through the nucleophilic attack of the 3′-OH of the guanosine in m^7^G in the α-phosphate of the next nucleoside triphosphate specified by the DNA template when the mRNA is capped and generates m^7^GpppGpG.^119^ Notably, m^7^GpppGpG is formed when this attack occurs on the 3′-OH of m^7^G, resulting in a reversed linkage, which causes approximately 50 percent of mRNAs to be capped in the reverse direction and cannot be recognized by the ribosome and hinders overall mRNA translation activity.^120–124^ Generally, anti-reverse cap analogs are synthesized to modify the m^7^G part of caps at the 2′ or 3′ position (2′-O-Methyl, 3′-O-methyl, 3′-H), which initiates exclusive cap incorporation in the correct direction and enhances translation efficiency.^125^

Poly(A) tails of IVT mRNAs are normally encoded in the DNA template or attached to IVT mRNA by enzymatic polyadenylation, and the former has more precise control of the length of poly(A) tails.^2,46^ Notably, a type II restriction enzyme for linearization of the plasmid template was used to contribute to an overhang at the 3′ end of the poly(A) tail when the poly(A) tail stretch was encoded in the template vector, which hampered the translational efficacy of IVT mRNA. This needs to be avoided by replacing the type II restriction enzyme with type IIS restriction enzymes.^46,126^

#### mRNA purification

IVT mRNAs are mixed with RNA polymerase and DNA templates after synthesis; thus, it is essential to purify IVT mRNA, including removing immunostimulatory contaminants, free ribonucleotides, short mRNA and DNA templates.^127^ Generally, DNase is used to degrade excess DNA templates. Commercial purification kits are often used to purify and separate the synthesized mRNA, followed by precipitation using ethanol or isopropanol, which can remove most contaminants and obtain high purity mRNA, and then the mRNA is precipitated with high concentrations of LiCl or alcohol-based precipitation, chromatographic methods (molecular exclusion chromatography, ion-exchange chromatography, or affinity chromatography with immobilized oligo-dT), or elution from a silica membrane column, which removes proteins, free nucleotides or other components but not dsRNA impurities.^128^ To remove dsRNA contaminants from the transcription reaction solution, Kariko et al. used reversed-phase HPLC to purify mRNA, which contributed to a dramatic increase in protein expression by 1,000-fold and completely eliminated the immune response of modified mRNA. However, it is unsuitable for scalable or larger mRNA production.^108,129^

RNase III, a novel purification method, has been proposed to eliminate dsRNA contaminants and has been shown to significantly reduce the immunogenicity of mRNAs and increase the cytotoxic killing efficacy of CAR T cells by electroporation of RNase III into CAR T cells. The potential drawback is that RNase III may cleave the double-stranded secondary structure formed by single-stranded RNA.^130^ Recently, cellulose chromatography was proposed to purify IVT mRNAs from micrograms to milligrams and produce large mRNAs up to 4 kb without any special equipment or toxicity, and its materials are all disposable, which poses no risk of cross-contamination compared to HPLC. Furthermore, cellulose chromatography showed higher efficiency in recovering and purifying IVT mRNA. Finally, short RNAs can be removed by denaturing polyacrylamide gel electrophoresis, and long RNAs can be separated by denaturing agarose gel electrophoresis.^108,131^ In summary, a variety of methods may be chosen to purify mRNA with different purity requirements and scales, which should be decided by the purpose of the research or application. Apparently, regardless of the method used for purification, strict mRNA quality control standards are the core to ensure the maximum benefits of mRNA therapeutics.

## mRNA delivery systems

Researchers initially demonstrated a negative attitude to the therapeutic potential of mRNA due to its instability in early explorations.^132^ mRNA delivery remains a great challenge for current mRNA-based therapeutics. Primarily, mRNA, as a negatively charged macromolecule (approximately 1–15 kb), has difficulty crossing the anionic cell membrane.^13^ Second, the median intracellular half-life of mRNA is only approximately 7 h.^133^ Furthermore, large amounts of mRNA are trapped in endosomes after entry and are unable to leak into the cytoplasm to exert translation functions, although naked mRNA is difficult to internalize via scavenger-receptor mediated endocytosis.^134^ Suitable delivery systems are required to achieve ideal mRNA potency, provide mRNA with protection and facilitate its cellular uptake as well as endosome escape, such as liposomes and polymers. Likewise, it should have low toxicity and immunogenicity.^135^ Inspiringly, mRNA can be accurately delivered to hepatocytes, Kupffer cells, and endothelial cells in the liver.^8^

**Fig. 6:**
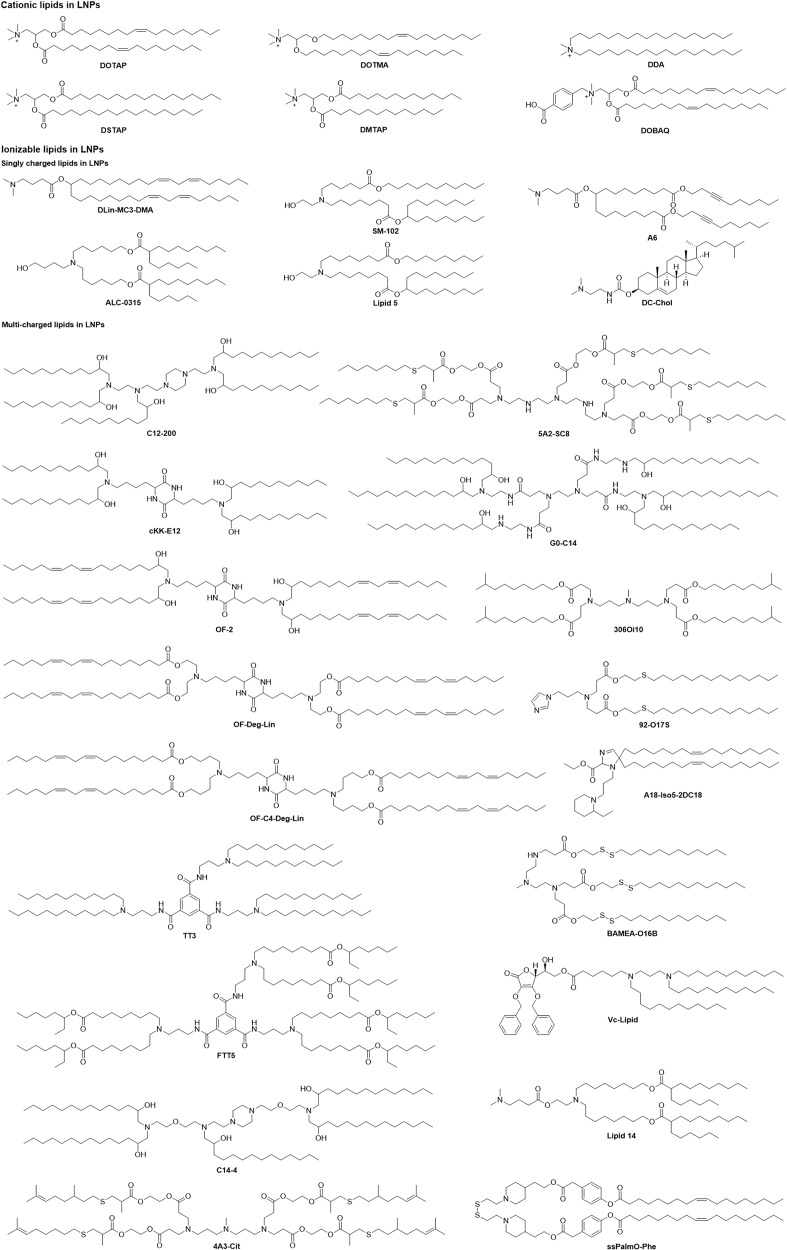
Positively charged lipids in mRNA-loaded lipid nanoparticles. The most widely used carrier of mRNA preparations is LNPs. Positively charged lipids play a vital role in LNPs because LNPs encapsulate mRNA through electrostatic adsorption between lipids and mRNA. These lipids can be classified into cationic lipids and ionizable lipids according to the generation of a positive charge. Furthermore, ionizable lipids can be divided into single-charged lipids and multicharged lipids. Here, we listed the representative lipids used in LNPs, including DOTMA, DOTAP, DSTAP, DMTAP, DDA, DOBAQ, DC-Chol,^8,171^ DLin-MC3-DMA,^540^ SM-102,^67^ A6,^163^ ALC-0315,^541^ and Lipid 5.^151^ Multicharged lipids in LNPs include C12-200,^512^ 5A2-SC8,^166^ cKK-E12,^542^ G0-C14,^151^ OF-2,^157^ 306Oi10,^154^ OF-Deg-Lin,^158^ 92-O17S,^160^ OF-C4-Deg-Lin,^543^ A18-Iso5-2DC18,^165^ TT3,^544^ BAMEA-O16B,^545^ FTT5,^546^ Vc-Lipid,^546^ C14-4,^161^ Lipid 14,^287^ 4A3-Cit,^547^ and ssPalmO-Phe^548^

mRNA-loading mechanisms likely involve electrostatic interactions, hydrogen bonds, or coordination interactions by thin-film hydration, nanoprecipitation, or microfluidic mixing. To enhance mRNA delivery, various vectors have been designed and synthesized, including LNPs, polymetric nanoparticles, cationic nanoemulsions (CNEs), and other delivery systems^136^ (Fig. 6). Optimization of mRNA delivery systems would significantly improve mRNA transfection efficiency and activity, which are integral steps for the development of mRNA drugs. Yang et al. constructed LNPs using cholesterol with modification of cationic peptide DP7 (VQWRIRVAVIRK), which improved intracellular mRNA delivery and the immune stimulation of DCs.^137^ Wang et al. used graphene oxide and polyethyleneimine (PEI) to form an injectable hydrogel, which carried mRNA-encoding ovalbumin and the adjuvant R848. The mRNA vaccine inhibited tumor growth in the B16-OVA melanoma model.^138^ Phua et al. used a mesoporous-silica nanoparticle to encapsulate mRNA and the inhibitor of RNA-activated protein kinase, C16. C16 enhanced the translation of mRNA, and the vaccine significantly inhibited tumor growth.^139^ Huang et al. utilized mRNA encoding a constitutively active mutation of the stimulator of stimulator of interferon genes (STING), which amplified the immune response induced by mRNA vaccines.^140^

Due to the extensive literature, we only briefly introduce the current developments in mRNA delivery vectors. We listed some typical vectors that bind mRNA with different interactions and form formulations by different preparation methods and summarized delivery vectors and adjuvants, payload mRNA, transfection efficiency, disease model or indication(s), routes of administration, and barriers to mRNA delivery.

### Lipid nanoparticles

#### Cationic lipid nanoparticles

Cationic lipids have been broadly used in mRNA delivery, including N-[1-(2,3-dioleoyloxy)propyl]-N,N,N-trimethylammonium chloride (DOTMA), 1,2-dioleoyloxy-3-trimethylammonium propane chloride (DOTAP), 1,2-stearoyl-3-trimethylammonium-propane (DSTAP), and 1,2-dimyristoyl-3-trimethylammonium-propane (DMTAP).^141^ Co-delivered mRNA and gardiquimod by a poly (lactic-co-glycolic acid, PLGA) -core/DOTAP-shell hybrid nanoparticle vector not only improved mRNA transfection efficiency but also aroused a strong immune response in the spleen and thereby inhibited tumor growth in mice with B16-OVA melanoma tumors.^142^ The research showed that using cationic lipids dimethyldioctadecylammonium (DDA), DOTAP, DMTAP, DSTAP, N-(4-carboxybenzyl)-N,Ndimethyl-2,3-bis (oleoyloxy) propan-1-aminium (DOBAQ) and 3ß-[N-(N’,N’-dimethylaminoethane)-carbamoyl] cholesterol (DC-Chol) in combination with 1,2-dioleoyl-sn-3-phosphoethanolamine (DOPE) to form LNPs delivered RVG mRNA, including inducing strong humoral and cellular-mediated immune responses in mice.^143^ DOTAP/Chol/DSPE-polyethylene glycol (PEG) cationic liposomes were employed to encapsulate cytokeratin 19 mRNA that provoked a strong cellular immune response and inhibited tumor growth in an aggressive Lewis lung cancer model by intranasal immunization.^144^ DOTAP liposomes modified with mannose targets were used to evoke humoral and cellular immune responses to treat the H1N1 influenza virus.^145^ The tremendous advantages associated with lipid-nanoparticle-based mRNA delivery systems, including their high stability, transfection efficiency, efficacy, safety, and low-cost manufacturing processes, have allowed the development of mRNA vaccines and drugs at unprecedented speed, and provide a powerful disease-fighting tool.^146^

#### Ionizable lipid nanoparticles

The ionizable amino lipid Dlin-MC3-DMA (MC3) has been used to deliver siRNA clinically for the treatment of transthyretin-mediated amyloidosis. Further research showed that the compound prescription of MC3 and lipidosis (DSPC, cholesterol, DMG-PEG2000, and DSPE-PEG2000) was applied for the delivery of IL-10 mRNA as an inflammatory bowel disease therapeutic, which expressed the anti-inflammatory cytokine IL-10 in Ly6c^+^ inflammatory leukocytes and alleviated symptoms in a dextran sodium sulfate colitis model.^147^ Correcting the genetic variance of cystic fibrosis transmembrane conductance regulator (CFTR) is an efficacy target to cure cystic fibrosis. Robinson et al. loaded CFTR mRNA in an MC3 delivery system into patient-derived bronchial epithelial cells and rescued the primary function of CFTR as a chloride channel.^148^ Clinically relevant LNPs composed of MC3, DSPC, cholesterol, DMG-PEG2000, and mRNA were transfected into 30 cell lines, and these data demonstrated that different transfection efficacies of different cell lines depended on an early and narrow endosomal escape window.^149^ Li et al. also employed MC3 LNPs covalently conjugated with αPV1 antibody-encapsulated mRNA to specifically target the lung by binding plasma vesicle-associated protein.^150^

Sabnis et al. developed and synthesized a new series of amino lipids similar to MC3 for delivering mRNA efficiently after single and repeat dosing by introducing ester linkages in the lipid tails and changing the position of ester linkages to achieve optimal chemical stability, tissue clearance, and mRNA delivery efficiency.^151^ Kimberly et al. synthesized ionizable lipids with high tolerability and reduced innate immune stimulation for mRNA by i.m. administration, these data indicated that different administration routes would result in different protein expression.^152^ In addition, degradable or nondegradable lipoids have been designed and investigated for intravenous or local delivery of mRNA to targeted tissues and cells. A small library lipoid using 3,3′-diamino-N-methyldipropylamine was designed to react with 11 saturated alkyl acrylate tails varying in length from 6 to 18, showing that the lipoid 306Oi10 with a one-carbon branch in the tail conferred a tenfold improvement over the lipoid 306O10 with the straight tail, whose nanoparticle-containing 306Oi10 efficacy ionizes at endosomal pH 5.0, thereby benefiting mRNA delivery.^153^ Both mRNA and siRNA were encapsulated in a lipoid nanoparticle composed of 306Oi10, cholesterol, DSPC, DOPE, and PEG-lipid, whose codelivery of mRNA and siRNA not only can improve improved gene silencing of siRNA but can also facilitate protein expression of mRNA.^154^

Nanoparticles containing cKK-E12 and nine different cholesterol variants were prepared for delivering mRNA, and the results revealed that the oxidative position of cholesterol influences nanoparticle targeting by adsorbing different protein coronas onto LNPs and that nanoparticles including 20α-OH cholesterol can target the liver.^155^ In addition, the cKK-E12 delivery system protected trastuzumab mRNA from degradation and enabled efficient in vivo delivery, which significantly delayed the growth of HER2-positive breast cancer.^156^ OF-02, which was obtained by altering the lipid tails of cKK-E12, produced twofold higher erythropoietin than cKK-E12.^157^ OF-Deg-Lin, an ionizable lipid that changes the local structure of OF-02 from 1,2-amino-alcohol to degradable ester linkage, delivers mRNA into the spleen, inducing protein expression in the B cell population.^158^ OF-C4-Deg-Lin was synthesized by altering the carbon linker lengths of OF-Deg-Lin specifically targeting the spleen.^159^ It is well known that most mRNA delivery systems have low transfection efficacy in primary T lymphocytes. The imidazole-based lipoids that were screened from a library of lipidosis combinations of amine heads and degradable tails containing S/S-S/Se/Se-Se could deliver mRNA into primary T lymphocytes.^160^

Similarly, a series of piperazine-centered compounds were synthesized and selected as CAR mRNA vectors for primary human T cells.^161^ For novelty, a battery of cationic lipid-modified aminoglycosides centering on commercially available aminoglycosides were synthesized to specifically deliver Luc mRNA to the liver.^162^ Many degradable and biocompatible cholesterol derivatives (OCholB lipids) containing disulfide bonds in the tail were constructed to target the lung and spleen.^163^ Likewise, lipidomic materials (A1-A6) containing alkyne and ester groups in the tails were obtained by changing the structure of Dlin-MC3-DMA to increase the tumorigenicity and facilitate endosomal escape, which co-formulated lipidomic materials to efficiently treat renal anemia.^163^ An ionizable LNP that was based on iBL0713 lipid for delivering EPO mRNA demonstrated comparable efficacy to Dlin-MC3-DMA-based formulations in the liver.^164^

### Lipid nanoparticles with immunostimulatory potency

Miao et al. developed lipidoses with cyclic amino head groups that activate the intracellular STING pathway, and LNPs composed of STING-activatable cyclic lipoids and OVA mRNA significantly prolonged survival and enhanced antitumor efficacy.^165^

Using 5A2-SC8-based dendrimer LNPs to encapsulate therapeutic FAH mRNA to produce FAH protein significantly increases the survival rate of FAH knockout mice suffering from HT-1.^166^ Choosing C12-(2-3-2)-based LNPs to encapsulate mRNA encoding angiotensin-converting enzyme 2 (ACE2) significantly improved liver and lung fibrosis.^62^ A redox-responsive NP platform consisting of G0-C14, a hydrophobic redox-responsive cysteine-based poly(disulfide amide) (PDSA), and lipid-PEG was used to deliver mRNA encoding p53, a critical tumor suppressor gene, to treat HCCs and non–small cell lung cancers (NSCLCs).^167^ A series of SS-cleavable proton-activated lipid-like materials based on vitamin E have also been applied to deliver mRNA to brain neuronal cells and astrocytes.^168^ Furthermore, TT3 lipid-like nanoparticles (TT3 LLNs) were used to codeliver mRNA and MRI contrast agents.^169^ Similarly, a theragnostic dendrimer-based LNP system formulated 4A3-SC8, pH-responsive PEGylated BODIPY dyes (PBD)-lipid and PBD were constructed for delivering mRNA and expressing protein in the liver, which was a promising delivery system for diagnosing and treating liver diseases and cancer.^170^

### Polymetric nanoparticles

Polymeric compounds and their derivatives can be synthesized from natural or synthetic materials, allowing for a wide variety of possible structures and characteristics.^171^ PEI is one of the most potent nonviral vectors for gene delivery. However, PEI is highly toxic and nonbiodegradable, limiting its application, so PEI-g-PEG with different PEG terminal groups and PEG grafting degrees were synthesized and achieved satisfactory potency for the delivery of mRNA to the lung.^172^ Dunn et al. also showed the polymers PEI1800-LinA5-PEG0.3 by modifying PEI-encapsulated mRNA and targeting the pulmonary microvascular endothelium.^173^ Poly (β-amino esters) (PBAE), a biocompatible and biodegradable polymer, were synthesized and used to deliver mRNA to target lung endothelium and pulmonary immune cells.^174^ A series of oligopeptide end-modified PBAEs (OM-PBAEs) with endosomal escape and cytoplasm penetration functions for transfecting mRNA were applied for specific liver tissue targeting.^175^ Polymers of hyperbranched poly (beta-amino esters) (hPBAEs) were applied to deliver mRNA to the lung epithelium via inhalation and produced sufficient protein in the lung with safety and compatibility.^176^ Similarly, a novel PCL-based PBAE was constructed to deliver mRNA into the spleen via intravenous injection.^177^ APE LNPs can deliver mRNA into the lung endothelium, liver hepatocytes, and splenic antigen-presenting cells (APCs) with high transfection efficiency.^177^ Charge-altering releasable transporters (CARTs), a kind of cost-efficiency and biodegradable polymer, were initially positively charged polymers that can load mRNA efficiently and improve physical properties through a degradative, charge-neutralizing intramolecular rearrangement, thus releasing functional mRNA and translating protein in cells.^178^ CARTs applied for mRNA delivery not only target professional APCs but also target local APCs.^179^ CARTs were employed to deliver mRNA that (coding costimulatory and immune-modulating factors, including OX40 L-, CD80-, and CD86-encoding) significantly inhibited tumor growth in both A20 and CT26 tumor models.^180^ Moreover, Schumann adopted PEG[Glu(DET)]2 polymer protected and delivered FS-344 mRNA that could express FS-315 follistatin protein to cure muscle atrophy via subcutaneous administration.^181^ A series of amphiphilic polyaspartamide derivatives PAsp (DET/R) were synthesized to deliver mRNA to Ai9 mouse brains via intracerebroventricular and intrathecal injection.^182^ PEG polyamino acid block copolymer PEG-PAsp (DET) was designed to deliver brain-derived neurotrophic factor mRNA to treat spinal cord injury with satisfactory recovery.^183^ In addition, some peptide-derived materials were used to deliver mRNA. For instance, PEG12KL4/mRNA complexes were formulated into dry powder by spray-drying and spray freeze-drying techniques for intratracheal administration;^184^ RALA, a cell-penetrating peptide, was applied to deliver antigen-encoding mRNA to the immune system.^185^ An advanced lip polyplex containing TriMan-lip (a trimannosyl diether lipid), Lip1, Lip2, and PEG HpK was developed to deliver mRNA to inhibit tumor growth and prolong the survival of mice.^186^

### Cationic nanoemulsion

CNEs were proposed as a potential nucleic acid delivery system in 1990^187^ and thus far have been proven to effectively deliver nucleic acids for the treatment of various diseases. The addition of cationic lipids to the formulation is essential for nucleic acid complexation through electrostatic interactions, which is also essential to improve the stability and transfection efficiency of nucleic acids and protect them from degradation by nucleases.^188^ Research shows that the self-amplifying mRNA (saRNA) CNE delivery system enhanced the local immune environment by recruiting immune cells and induced cellular responses to antibodies and T-primates at relatively low doses (75 µg).^189^

### Other mRNA delivery systems

Other types of vectors were developed to deliver mRNA, including protamine-condensed mRNA, exosomes, extracellular vesicles (EVs), mesoporous silica, CaP and so on.^190^ Reactive astrocyte-derived exosomes were used to deliver MGMT mRNA to MGMT-negative glioma cells and inhibited temozolomide resistance.^181^ EVs with a high-affinity anti-HER2 scFv antibody (ML39) were also applied to deliver HchrR6 mRNA to recipient cells and tumors.^191^ Tetrasulfide-incorporated large-pore dendritic mesoporous organosilicon nanoparticles were constructed to consume intracellular GSH, thereby enhancing mRNA translation.^192^ Lipid-coated calcium phosphate NPs containing CaP core, DOPA, DOTAP, and DSPE-PEG for delivering MUC1 mRNA with anti-CTLA-4 monoclonal antibody were designed to treat triple-negative breast cancer.^191,193^ Nucleoside lipids for delivering mRNA have attracted public attention because mRNA can be loaded inside lipids through the hydrogen bonding interaction of base complementary pairings with good compatibility and safety. Uchida et al. hybridized a PEG-conjugated oligonucleotide (PEG-oligoRNA) with mRNA through hydrogen bond complementarity (20:1) to obtain PEGylated mRNA, which was then loaded with Lipofectamine LTX, and the delivery system maintained a high degree of structural stability in vivo.^194^ Polyplex micelles were developed by combining ω-cholesteryl (ω-Chol)-poly (ethyleneglycol) (PEG)-polycation block copolymers with mRNA prehybridized with cholesterol (Chol)-tethered RNA oligonucleotides (Chol (^+^)-OligoRNA) to improve the tolerance of mRNA nucleases and the stability of mRNA.^195^ Furthermore, an RNA linker that connected 10 nt oligo-adenine nucleotides (OligoA) with two 17 nt oligonucleotides was designed to improve the stability of mRNA to ribonuclease.^196^ Generally, most of the reported delivery vectors deliver mRNA through electrostatic interactions or hydrogen bond interactions. Novel delivery vectors have also emerged for further application, such as self-assembled core–shell nanoscale coordination polymer nanoparticles that were used to deliver siRNA, microRNA or DNA through coordination interactions.^197–199^ Overall, among mRNA delivery platforms, LNPs have been approved for clinical use and have been shown unique advantages, and potential nanomaterial candidates are still emerging. The choice of mRNA delivery system depends on the size of the delivered mRNA molecule, the charge, and the organ to be targeted. There are advantages and disadvantages to different delivery materials.

### In vitro and in vivo barriers to mRNA delivery

It has always been the focus of our thinking by increasing cell uptake, facilitating lysosomal escape, and speeding up translation to maximize the availability of mRNA.^200^ Nanoparticle-based delivery systems provide a promising approach to improve cell uptake and lysosomal escape, which are also widely researched in the field of mRNA delivery.^201^ Multiple steps are involved in mRNAs entering the cytoplasm with the help of nanoparticles: endocytosis, lysosomal escape, and mRNA release. The cell membrane is a dynamic and formidable barrier to intracellular transport.^201^ Nanoparticles interact with cell membranes through various mechanisms, including clathrin-dependent endocytosis, caveolae-dependent endocytosis, and micropinocytosis,^202^ so particle properties, including particle shape, size, material composition, and surface charge, are involved in cellular uptake.

It is a prerequisite for efficient mRNA delivery to comprehend the mechanism of mRNA cellular uptake. It has been reported that naked mRNA is internalized by scavenger receptors without delivery materials and subsequently accumulates in lysosomes; minimally, mRNA escapes into the cytosol and expresses proteins, so it is necessary to use vectors for the intracellular delivery of mRNA and overcome the initial energy barrier to mRNA uptake.^203^ Stimulating scavenger receptor activity to increase the uptake of mRNA and promoting endosomal escape could boost the availability of mRNA in the cytoplasm.^204^ mRNA needs to be released from lysosomes and egressed to cytosol to translate encoding protein and was inevitably inhaled to lysosomes following micropinocytosis and clathrinid-mediated endocytosis, where acidic and enzyme-rich environment is prone to degradation of nanocarrier and mRNA, so lysosome degradation is another delivery barrier for mRNA.^205^ At present, electroporation is used for clinically delivering mRNA ex vivo, but its disadvantage is that membrane destruction by electroporation may lead to the loss of cytoplasmic content with significant cytotoxicity.^206^

Notably, endosome/lysosome formation is essential for exogenous mRNA function because the mammalian target of rapamycin on the lysosomal surface involves several cellular processes, including protein expression and mRNA transfection efficiency. The rapid rate at which nanoparticles are engulfed by lysosomes is directly affected by the properties of nanoparticles, so as quickly as possible to escape lysosomes is necessary for mRNA translation.^149,207–209^ Nanoparticle materials achieve lysosome escape through conductivity, such as DOPE, MM27, and DLinDMA, which are widely applied to the cell membrane in an acid-mediated manner.^200,201,210^ In addition, pH-responsive cell-penetrating peptides promoted endosome membrane disruption and enhanced protein expression.^211^ Recently, research showed preassembling an mRNA translation initiation structure called ribonucleoproteins through an intrinsic molecular recognition between m^7^G-capped mRNA and eIF4E protein, thereby mimicking the first step of intracellular protein synthesis, and subsequent ribonucleoproteins electrostatically stabilized with structurally adjustable cationic carriers to form nanoplexes. This approach significantly improved mRNA transfection efficiency by enhancing intracellular mRNA stability and protein synthesis.^200^ Collectively, engineering precision nanoparticle delivery systems for mRNA-based therapeutics is the key to determining mRNA translation efficiency and enhancing the expression of mRNA.

There is also a substantial challenge for mRNA delivery in vivo.^212^ Nude mRNA is directly used for mRNA-based therapeutics; however, it is vulnerable to the widely distributed RNase in vivo. Therefore, a delivery system is essential for mRNA-based therapeutics.^213^ Research on siRNA vectors is relatively mature. Regrettably, these vectors for siRNA and pDNA delivery may be unsuitable for mRNA delivery owing to their different characteristics.^214^ Therefore, it is urgent to develop new delivery vectors to achieve favorable loaded mRNA circulation, specific target organs or cells, cytomembrane penetration, lysosome escape, and mRNA and protein expression.^215^

There have been many reports on the enhancement of mRNA encoding antigen uptake by DCs through cell receptor modification of nanoparticles.^208^ There are still numerous barriers to uptake and intracellular trafficking that determine mRNA-based therapeutic efficiency.^216^ DCs play key roles in immunotherapy, which can efficiently take up, process, and present antigens and subsequently induce humoral and cellular immunity against various infectious diseases and cancers.^217^ DC-based vaccines are a potent immunotherapeutic strategy. Autologous DCs are used to load antigens by pulsing in vitro and are then administered back to the patient to initiate the immune response.^218^ There are several strategies to deliver mRNA into the cytoplasm of DCs, including electroporation, lipofection, and sonoporation.^219^ Electroporation is possibly the most diffusely used method for mRNA introduction, which rapidly introduces tumor-associated antigen (TAA)-encoding mRNA by using a relatively weak electric pulse, greatly avoids the degradation of mRNA by ubiquitous extracellular ribonuclease, and mediates mRNA cellular processing and presentation on the DC surface.^220^ Lipofection encapsulates and delivers mRNA into DCs by forming mRNA lipoplexes, which are subsequently taken up via cell endocytosis, and then the lipid fuses with the endosomal membrane to release mRNA into the cytoplasm.^221^ For the sonoporation strategy, mRNA is loaded in microbubbles and directly crosses the cytoplasm membrane via temporary pores, which are created by oscillating microbubbles and imploding them using ultrasound.^222^ The transfection and expression efficiency of mRNA drugs in DCs is the key to therapeutic efficacy. Different delivery strategies contribute to distinct mRNA transfection efficiency, namely, electroporation (90%), lipofection (5–50%) and sonporation (5–50%).^223–225^ Importantly, electroporation has high transfection efficiency and is used to treat various tumors in clinical studies, including melanoma,^226–228^ AML,^76^ ovarian cancer, and infectious diseases (human immunodeficiency virus [HIV]).^229^ In addition, previous research showed that lipofection provides the high expression of antigen and is more effective in expanding CD8^+^ T cells in DCs, indicating that lipofection has potent immune stimulation activity. However, the reproducibility of transfection efficiency makes GMP-standard manufacture implementation difficult and restricts lipofection clinical application.^230^ Collectively, focusing on optimized delivery strategies that overcome DC barriers is the key to mRNA-based immunotherapy.

The in vitro and in vivo efficiency of mRNA drugs is not always consistent. The transfection efficiency of alkyne lipids outperformed MC3, cKK-E12, and C12-200 in vitro but not in vivo.^163^ In addition, encapsulation of different mRNAs delivered extracellular displayed different distributions; OF-Deg-Lin LNPs loaded with Cy5 mRNA were transported predominantly to the liver, whereas OF-Deg-Lin LNPs encapsulated FLuc mRNA expressed protein in the spleen.^158^ We speculated that the abovementioned inconsistencies may be caused by the complicated internal environment, including the immune system, variable blood flow, heterogeneous vasculature, and off-target cells, and the specific mechanisms still need to be further explored.

Tissue-targeted delivery of mRNA-based therapeutics is essential for efficient in vivo delivery of mRNA.^67^ Delivery systems can provide much more effective and targeted delivery of mRNA drugs, including drug release that is triggered by the specific microenvironment and the physicochemical properties of mRNA vectors that play important roles in their systemic delivery and biodistribution.^231^ Engineering precision nanoparticles for mRNA-based drug delivery has expanded into a broad range of clinical applications and has been developed to navigate biological barriers.^171^ Nanoparticles are rapidly recognized by mononuclear phagocytic systems in the liver and spleen by binding to serum proteins, and encapsulated mRNA is released to target cells.^221^ The majority of the current most widely used mRNA-based delivery of LNPs specifically targets the liver, and LNPs continue to focus on optimizing delivery platforms in other tissue-targeted delivery.^232^ Recently, selective organ targeting has emerged as a therapeutic strategy to precisely and predictably optimize LNPs and allow them to deliver mRNA and Cas9 mRNA/single guide RNA and Cas9 ribonucleoprotein complexes to target tissues via intravenous injection into the liver and lung.^233^ In addition, cell-targeted delivery of mRNA-based therapeutics, especially DCs and APCs, plays crucial roles in shaping immune responses by delivering requisite signals to T cells and activating expansion and differentiation T cells.^210^ The field of mRNA-based therapeutics is currently focused on the development of novel materials and formulations that can potentially enhance transfection efficiency and therapeutic efficacy.^2^

### The adjuvant activity of mRNA delivery systems

Cationic liposomes themselves act as adjuvants, and their main function is to protect the antigen from being eliminated and deliver the antigen to professional APCs.^234^ The RNActive (CureVac AG) vaccine platform relies on its carrier to provide adjuvant activity, and the adjuvant activity is provided by the codelivery of RNA complexed with protamine (a polycationic peptide) by inducing an adaptive response,^235–237^ which has elicited a favorable immune response in multiple preclinical animal studies against cancer and infectious diseases.^238–241^ Mechanistically, the adjuvant properties of the RNActive vaccine showed a potent TLR7/8-dependent immune response, including activation of TLR7 (in mouse and human cells) and TLR8 (in human cells), type I interference, cytokines, and chemokines.^235^ However, mRNA-mediated activation of type I interferon may cause protein translation and CD8^+^ T cell activation to be inhibited, which may be related to the kinetics of type I interferon signaling relative to TCR activation.^242,243^ The codelivery of mRNA and hydrophobic TLR7 adjuvant (gardiquimod) is achieved by a PLGA core/lipid-shell hybrid nanoparticle system, in which PLGA allows incorporation of the adjuvant into the nucleus and the lipid shell loads the mRNA through electrostatic interactions. The nanoparticle realizes a strong antigen-specific immune response and highly effective antitumor activity.^142^

### The effect of administration routes on delivery efficiency

The administration routes play a vital role in the mRNA delivery system because some specific diseases require specific routes of administration, although intravenous administration can meet the needs of most diseases. For instance, inhaled administration or intratracheal administration is suitable for pulmonary diseases;^184^ cerebral diseases may be cured by intracerebroventricular injection or intrathecal injection;^182^ and liver diseases may be treated via intravenous, intraperitoneal, subcutaneous, or intramuscular administration.^244^ In addition, different delivery vectors will have different distributions or expressions under different administration routes. For example, LNPs containing lipidoid 306Oi10 targeted and expressed protein predominantly in the liver via i.v. injection, while the LNPs accumulated in the pancreas (11%), kidneys (12%), and lungs (15%) and expressed protein in the liver (67%), pancreas (17%), and spleen (13%); similarly, the LNPs drained through capillaries and the lymphatic system when administered via s.c. and i.m.^244^ It has been reported that cholesteryl-based disulfide bond-containing biodegradable cationic liposome nanoparticles OCholB LNPs have demonstrated the successful delivery of mRNA molecules in the skeletal muscle (via intramuscular injection), lung and spleen (via intravenous injection), and brain (via lateral ventricle infusion).^162^ CARTs preferentially targeted professional APCs in secondary lymphoid organs upon i.v. injections and targeted local APCs upon s.c. injection.^179^ Therefore, the optimal therapeutic efficacy can only be achieved by selecting the appropriate mRNA delivery vectors and routes of administration. Collectively, LNP–mRNA therapeutics (good manufacturing practices, stability, storage, and safety) have great potential in the treatment of infectious diseases, cancer, and genetic diseases. The development of mRNA delivery systems with high efficiency and safety is of great significance for the wide application of mRNA-based therapeutics.

## Application

**Fig. 7:**
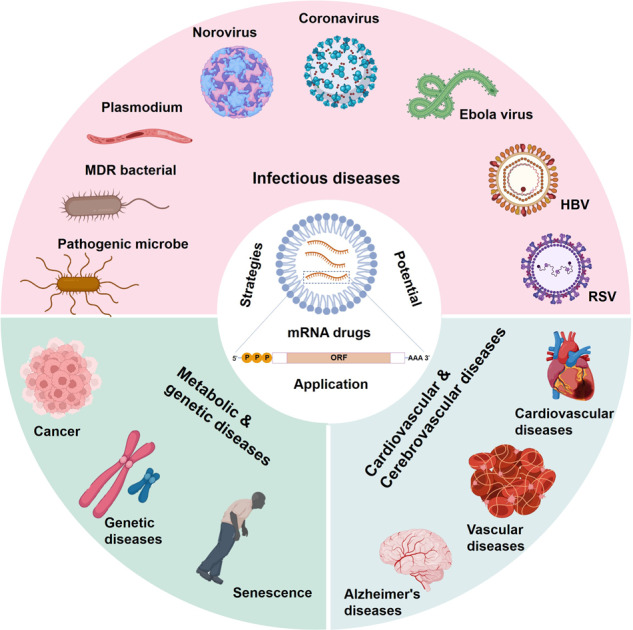
Strategies and potential application of mRNA-based therapeutics. mRNA drugs have yielded numerous inspiring treatments for refractory or previously incurable diseases, including infectious diseases, genetic diseases, cancers, and cardiovascular diseases. In particular, the mRNA vaccine has shown a strong advantage in the prevention of SARS-CoV-2 infection and may also be a potential approach against the infection of other viruses and pathogenic microorganisms, including malaria, respiratory syncytial virus, and HIV^13^

mRNA-based therapeutics are expected to become a powerful therapy for a variety of refractory diseases, including infectious diseases, metabolic genetic diseases, cancer, cardiovascular and cerebrovascular diseases, and other diseases (Fig. 7). A large number of studies have shown that mRNA cannot only mediate better transfection efficiency and longer protein expression but also has greater advantages than DNA and traditional protein drugs; mRNA initiates protein transient translation when reaching the cytoplasm without inserting into the genome, which has a lower insertion risk compared with traditional protein and DNA drugs. Importantly, mRNA is easily synthesized through the IVT process, is relatively easy to manufacture and can be quickly applied to various therapies. In addition, the two most concerning issues in mRNA, immunogenicity and stability, are controlled by the chemical modification of selected nucleotides. mRNA therapy has attracted billions of dollars, and an increasing number of well-funded biotechnology companies have been established, such as Moderna, CureVac, BioNTech, Argos Therapeutics, RaNA, Translate Bio, Ethris, Arcturus, and Acuitas (Table 2). Apparently, mRNA has become one of the most attractive areas for drug development, which is definitely worth exploring in the long term. In this section, we comprehensively summarize the latest developments in the current state of mRNA-based drug technologies and their applications.

**Table 2:** Current status in mRNA therapeutics development

Therapeutic areas	Therapeutic strategy	Indication	Company
Infectious diseases	Vaccine	COVID-19	Moderna, BioNTech, Curevac, Sirnaomics, eTheRNA, Walvax, Translate Bio, Ethris, Arcturus, Tiba, Acuitas, StemiRNA, RNACure, Abogen, Precision NanoSystems, Longuide Limited Lab
		Influenza	Moderna, BioNTech, Curevac, Sirnaomics, Arcturus, Tiba, StemiRNA, RNACure
		RSV infection	Moderna, Curevac, Ethris, RNACure
		HIV infection	Moderna, BioNTech, eTheRNA, Argos
		Rabies	Curevac, Precision NanoSystems
		HPV infection	Sirnaomics, eTheRNA, StemiRNA
		Malaria	BioNTech, Curevac, eTheRNA
		EBV infection	Moderna, StemiRNA
		Tuberculosis	BioNTech, StemiRNA
		CMV infection	Moderna, Rhegen
		Herpes zoster	Abogen
		Zika virus infection	Moderna
		HBV infection	Sirnaomics
		Yellow fever	Curevac
		PIV infection	Moderna
		hMPV infection	Moderna
		Rotavirus infection	Curevac
		Nipah virus infection	Moderna
	Antibody	COVID-19	BioNTech, Sirnaomics
		Chikungunya virus Infection	Moderna
	Gene editing	HIV	Sangamo
Oncology	Vaccine	Melanoma	BioNTech, Curevac, eTheRNA
		NSCLC	BioNTech, Sirnaomics
		Cervical cancer	Sirnaomics, eTheRNA
		Breast cancer	Sirnaomics
		Ovarian cancer	BioNTech
		Liver cancer	Sirnaomics
		Gastric cancer	eTheRNA
		Pancreatic cancer	Sirnaomics
		Colorectal cancer	BioNTech, Sirnaomics
		Bladder cancer	Sirnaomics
		Prostate cancer	BioNTech
		Head and neck cancer	BioNTech, Curevac
		Adenoidcystic carcinoma	Curevac
		cSCC	Curevac, Sirnaomics
		Basal cell cancer	Sirnaomics
		Renal cell cancer	eTheRNA, Argos
		AML	StemiRNA
	Personal vaccine	Ambiguity	BioNTech, Argos, StemiRNA, RNACures, Rhegen, Abogen
	CAR-T	Pancreatic cancer	BioNTech,
	Antibody	Pancreatic cancer	BioNTech,
Genetic diseases	Protein replacement	Cystic fibrosis	Moderna, Translate Bio, Arcturus
		Propionic acidemia	Moderna
		Methylmalonic acidemia	Moderna
		GSD1a	Moderna
		Phenylketonuria	Moderna
		CN-1	Moderna
		OTC	Arcturus
		Hemophilia	Sirnaomics
Autoimmune disorders	Protein replacement	Ambiguity	Moderna, eTheRNA, Tiba
Metabolic disorders	Protein replacement	Type 2 diabetes	Moderna
Cardiovascular disease	Protein replacement	Hypercholesterolemia	Sirnaomics
		Myocardial ischemia	Moderna
Fibrosis	Protein replacement	Hypertrophic scarring	Sirnaomics
		Liver fibrosis	Sirnaomics
		Lung fibrosis	Sirnaomics
		Primary sclerosing cholangitis	Sirnaomics
		Anemia	In-Cell-Art

### mRNA therapeutics that are directly based on the encoding molecules

The aforementioned mRNA-based immunotherapy achieves promising outcomes by expressing antigens and then initiating immune responses,^245^ which is defined as an indirect therapy that does not target the virus or tumor cells with mRNA encoding therapeutic proteins.^246^ mRNAs encoding proteins/peptides directly target viruses, bacteria, or cancer cells. In contrast, mRNA therapeutics directly treating diseases by delivering mRNA-based functional proteins are considered a direct strategy, including missing or downregulated endogenous proteins, functional foreign proteins or antibodies, and proteins for gene editing tools.^247^ In addition, the strategy of directly expressing proteins in “cell factories” can also be used to engineer cells, such as engineered T cells.^161^ mRNA-based protein replacement therapeutics have already entered the clinical stage despite the limited number of clinical trials vs. mRNA vaccines.^248,249^

#### mRNA-based monoclonal antibodies

Antibody-based drugs have achieved rapid progress in biopharmaceutics, but the worldwide application of monoclonal antibodies (mAbs) is limited by their vulnerable properties and the high cost of production, storage, transportation, and distribution.^250^ Nucleic acid-encoded mAbs, especially mRNA-based monoclonal antibodies, have rendered great hope for improving antibody therapy efficacy, and targeted cells are expropriated as factories to translate nucleic acids into functional mAbs.^251^ Plasmid DNA-encoded mAbs are usually concentrated in the area of infectious diseases, and some have already entered the clinical stage, while studies on mRNA-based mAbs (mRNA-mAbs) have relatively lagged. Here, we focus on the application of mRNA-mAbs, which are mostly concentrated on the treatment of infection and tumors.^252^ The broadly neutralizing anti-HIV-1 antibody VRC01 was decoded into nucleoside-modified mRNA, and systemic administration of the LNP-encapsulated mRNA successfully produced VRC01 at the efficacy level and protected humanized mice from intravenous HIV-1 challenge.^253^ For human RSV, Tiwari et al. developed the existing drug palivizumab into engineered mRNA encoding membrane-anchored neutralizing antibodies, which displayed higher efficiency than palivizumabs and significantly inhibited RSV 7 days post-transfection.^254^ Isolated neutralizing mAbs (CHKV-24) from the B cells of a survivor of natural chikungunya virus infection were successfully encoded by mRNA, expressed at biologically significant levels in vivo, and protected mice from arthritis and musculoskeletal tissue infection with reduced viremia at undetectable levels after 2 days of inoculation.^255^ A nanostructured lipid carrier was exploited to transfer replicon RNA encoding ZIKV-117 mAb in situ by intramuscular delivery, which contributed to high levels of mAb expression and protected mice from lethal ZIKV infection.^256^ In addition, the strategy of mRNA-based mAbs is adopted in the treatment of tumors. Various mRNA-based antibodies against cancer were designed and induced rapid and sustained serum antibody titers in vivo, which allowed mice to survive the challenge of non-Hodgkin’s lymphoma tumor incubation.^257^ Anti-HER2 antibody (trastuzumab) was systemically delivered using IVT mRNA LNPs and synthesized in vivo, which improved the pharmacokinetic profile in comparison with directly injecting trastuzumab protein.^156^ In addition, Zhou et al. reported a novel method for rapidly delivering the nanobody/variable domain of the heavy chain from an antibody by introducing its coding mRNA.^258^ Bispecific T cell-engaging antibody (bsAb) has emerged as a promising approach to treat malignancy, although this is somewhat impeded by manufacturing difficulty and short serum half-life. Endogenously synthesized and durable bsAbs through systemic administration (mRNA-based bsAbs) efficiently inhibited tumor growth.^259^ Ye et al. developed a saRNA encoding an anti-SARS-CoV-2 antibody with an alphavirus vector.^99^ However, the virus vector showed poor safety in the development of the SARS-CoV-2 mRNA vaccine.^260,261^

### mRNA-based immunotherapy

**Fig. 8:**
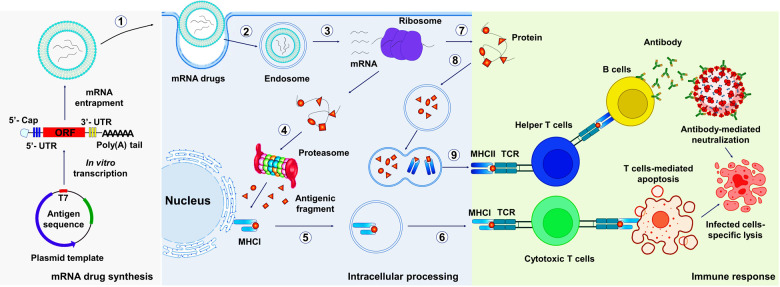
mRNA drugs elicit immunity using disease-specific targeting antigen strategies. mRNA drugs mainly go through the following three aspects from synthesis to initiate immune protection, including mRNA synthesis, intracellular processing, and initiating immune protection. Briefly, IVT mRNA drugs are encapsulated into carriers (such as nanoparticles) and are endocytosed by antigen-presenting cells (**①-②**); mRNA is released into the cytoplasm after escaping from endosomes and then translated into antigenic proteins by ribosomes (**③**). Subsequently, endogenous antigens are degraded into polypeptides by the proteasome and are presented by MHC I and activate cytotoxic T cells (CD8^+^ T cells) (**④-⑥**). In addition, secreted antigens can be taken up by cells, degraded inside endosomes, and presented on the cell surface to helper T cells by MHC class II proteins (**⑦-⑨**). Finally, helper T cells (CD4^+^ T cells) stimulate B cells to produce neutralizing antibodies against pathogens^382^

Immunotherapies have yielded numerous inspiring treatments for refractory or previously untreatable diseases, including infectious diseases, cancers, autoimmune diseases, and allergies.^262–266^ Vaccine research progress has fueled a great deal of enthusiasm and promise for immunotherapy approaches against pandemic infectious diseases, including attenuated vaccines, inactivated vaccines, and protein subunit vaccines.^267^ Recently, nucleic acid vaccines have emerged as innovative vaccines, including DNA vaccines and RNA vaccines. Notably, mRNA-based therapeutics have emerged as a safe and efficacious strategy to protect patients from infectious diseases and cancers due to their extraordinary advantages, including high efficiency, a relatively low severity of side efficacy, and ease of manufacture.^1,262^ Here, we reveiwed the applications of mRNA-based drugs, focusing on clinical trials of prophylactic and therapeutic vaccines for infectious diseases and cancers (Fig. 8).

#### mRNA vaccines against infectious diseases

##### SARS-CoV-2 mRNA vaccines

SARS-CoV-2 emerged in 2019^268^ and then caused pandemics worldwide.^269^ To date, there have been more than 228 million confirmed cases of COVID-19, including ~6.14 million deaths according to the WHO report (covid19.who.int). The first COVID-19 vaccine (Pfizer-BioNTech COVID-19 Vaccine; BNT162b2) was approved by the FDA for emergency use authorization and subsequently for the Moderna COVID-19 vaccine (mRNA-1273). These vaccines provide ~90% effectiveness prevention of infection for full vaccination and 80% for partial vaccination,^270–273^ However, neutralization antibodies against the SARS-CoV-2 Omicron variant are undetectable in the sera of most mRNA-1273 or BNT162b recipients, while additional mRNA vaccine dose seems to improve the neutralization.^274^

**Fig. 9:**
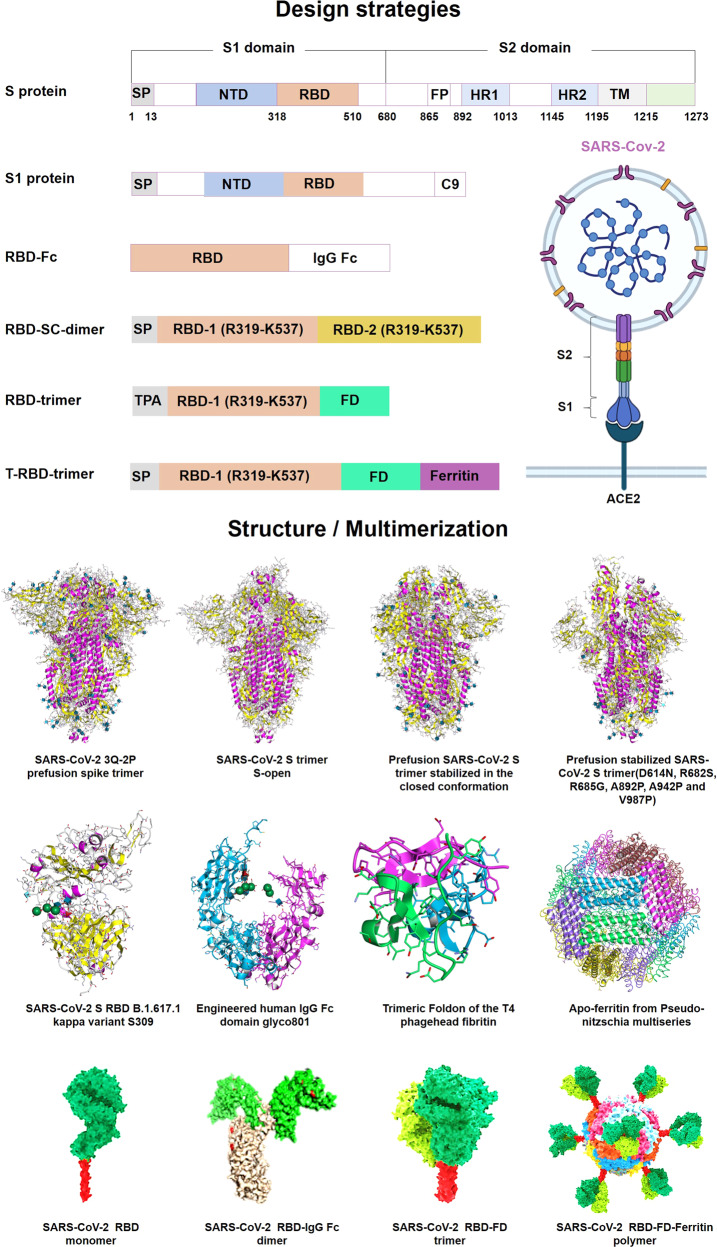
SARS-CoV-2 mRNA antigen immunogenicity and vaccine design. Full-length S-protein or RBD as a vaccine immunogen has been widely confirmed to induce high-affinity neutralizing antibodies. SARS-CoV-2 S protein is intrinsically metastable and can be stabilized in a prefusion conformation by structure-based design.^549,550^ Prefusion-stabilized SARS-CoV-2 Spike immunogen induces potent humoral and cellular immune responses.^551,552^ The RBD peptide is one of the most promising targets to design candidate vaccines. However, RBD has a low molecular weight, which leads to its weak immunogenicity, and can be further improved by forming multimers. Multimerization of RBD protein using humanized IgG Fc,^284^ T4 trimerization (FD)^285^ or Ferritin^286^ have been shown to induce higher neutralizing antibody compared to monomeric antigens, which will provide us with new ideas for designing powerful mRNA vaccines

SARS-CoV-2 consists of structural proteins, spike (S), nucleocapsid (N), envelope (E), and membrane (M).^275^ The coronavirus S protein or the RBD of the S protein mediates receptor binding and fusion of the viral and cellular membranes, and entry of virions into target cells has emerged as an antigen therapeutic strategy to design vaccines.^276^ N proteins of SARS-CoV-2 can induce immune responses to inhibit viral infection, while E proteins and M proteins are generally not taken into account for the lack of immunogenicity.^277,278^ Several strategies have been used to improve the COVID-19 vaccine effect; prefusion S protein was formed by mutation of two proline residues of the spike protein to stabilize it in the prefusion conformation, and BNT162b2 and mRNA-1273 both used 1-methyl-3′-pseudouridylyl modified mRNA (m1Ψ mRNA) encoding prefusion S protein.^271,279^ SARS-CoV-2 spike RBD, as the binding site for hACE2, facilitates virus entry into target cells and is a promising target to design candidate vaccines.^280^ However, monomeric RBD antigens have limited ability on engaging interactions with B cell receptors thereby facilitating the generation of high-affinity antibodies.^281–283^ Various strategies have been developed to increase RBD protein immunogenicity, thus enhancing antibody titers, including conformation dimers, trimers or polymers, by adding humanized IgG Fc,^284^ T4 trimerization (Foldon)^285^ or ferritin^286^ to antigen (Fig. 9). mRNA that encodes the C-terminal fold or Helicobacter pylori ferritin rendered a multimeric conformation of RBDs and induced robust and durable humoral immunity.^286^ mRNA encoding RBD-conjugated Fc induces a stronger immune response.^287^ Furthermore, mRNA drugs can also effectively block the binding of the RBD to the human ACE2 receptor by encoding high-affinity truncated ACE2 variants.^288^

Several SARS-CoV-2 variants have emerged with the global COVID-19 pandemic.^289^ Fortunately, chimeras of the viral S protein were developed to prevent SARS-CoV-2 variants,^270^ and BNT162b2 and mRNA-1273 can still effectively prevent SARS-CoV-2 variants infections, including *Delta* (B.1.617.2), *Alpha* (B.1.1.7) and *Gamma* (P.1) variants in adults.^272,290,291^ Interestingly, there is a large difference in the mRNA dosages of COVID-19 mRNA vaccines. The approved dosage of one dose of BNT162b2 is 30 μg mRNA, and mRNA-1273 is 100 μg (www.fda.gov/). The first 100 μg BNT162b1 vaccination lacked meaningfully increased immunogenicity compared with the first 30 μg vaccination.^292^ Nevertheless, dose-dependent responses were observed in the vaccinations of mRNA-1273 (25, 100, and 250 μg) and ARCoV (100 and 1000 μg).^276^ Notably, a saRNA vaccine encoding the S protein and the VEE virus replicase for self-amplification, called LUNAR-COV19, were designed and showed that a single 2 μg vaccination protected mice from lethal SARS-CoV-2 infection.^293^

The duration of the COVID-19 mRNA vaccine and its effectiveness in special populations necessitate further investigation into long-term protection, especially for patients with existing conditions and a pandemic pathogen with mutations. The anti-SARS-CoV-2 humoral immunity continuously declined for several months following full BNT162b2 or mRNA-1273 vaccination.^294–297^ BNT162b1 induced weaker humoral immunity in older adults than in younger adults.^285,298^ Fortunately, BNT162b2 vaccination appears to be safe for pregnant women and can reduce the risk of SARS-CoV-2 infection.^299–302^ Likewise, anti-SARS-CoV-2 antibodies can be transferred to neonates in pregnancy.^303^ BNT162b2 and mRNA-1273 appear to be well tolerated and induce a weaker but significant immune response in patients with immunocompromising conditions, including hemodialysis,^304^ hematological disorders,^305,306^ malignancy,^307,308^ chronic inflammatory disease^309^ and HIV infection (only BNT162b2 evaluated).^310^ BNT162b2 showed weaker but significant immunogenicity in patients with autoimmune diseases, including rheumatic diseases,^311–313^ multiple sclerosis,^311,314–316^ myasthenia gravis,^317^ and musculoskeletal diseases.^318^ Notably, mRNA-1273 and BNT162b2 showed impaired immunogenicity in solid organ transplant recipients.^319–323^

Various pathogens cause serious human infections, including viruses, bacteria, fungi, and parasites.^324^ Viruses have caused a series of public health emergencies: the H1N1 influenza pandemic in 2009–2010,^325^ Zika virus infection in 2015–2016,^326^ and the current COVID-19 pandemic.^327^ Vaccines are a vital tool in the battle against infectious diseases.^328^ mRNA vaccine candidates have shown similar safety and reactogenicity profiles to inactivated vaccines approved by the European Union and Americans, but acute and chronic infections account for 15% of all deaths worldwide due to unreasonable vaccine distribution in resource-limited areas and insufficient response to infectious outbreaks.^329^ mRNA vaccines are an ideal approach to overcome these challenges and fulfill the urgent need for vaccines during epidemics in a timely manner.^330^ Currently, mRNA vaccines have been intensively researched and developed to combat highly contagious SARS-CoV-2, influenza virus, Zika virus, rabies virus, and HIV, and corresponding clinical results are summarized (Table 3).^331^ mRNA vaccine candidates were rapidly generated 8 days after the publication of hemagglutinin and neuraminidase genes of H7N9 influenza virus. An mRNA vaccine (NCT03014089) showed 47% placentas from Zika virus infection in comparison with 91% infected placentas of placebo-vaccinated mice, and protective humoral immunity was also confirmed in rhesus macaques.^332^ Likewise, mRNA-1273 successfully decreased the viral load in the lungs of mice and rhesus macaques challenged with SARS-CoV-2 and evoked a Th1-biased immune response in healthy adults (NCT04283461, NCT04470427).^333^ An mRNA vaccine (CV7201) was developed by using mRNA encoding the glycoprotein of rabies virus to treat rabies, which showed temperature stability and successfully elicited a WHO-specified antibody response in >70% of participants via three rounds of intradermal (i.d.) vaccination (NCT02241135).^334^ Despite extensive efforts in design and testing, scientists failed to generate an effective preventive HIV vaccine. Unlike the prophylactic vaccines above, the mRNA vaccines for HIV not only aim to prevent but also aspire to cure infection. Anti-HIV mRNA vaccine (NCT02888756) and DC-based mRNA vaccine (AGS-004, NCT00672191) have entered clinical trials,^335^ but no antiviral efficacy has been observed in clinical trials.^336^ Vibcinated patients had similar plasma virus levels to placebo-treated controls (NCT00672191), and all participants restarted antiretroviral therapy for unsuccessful control of acute HIV infection (NCT00672191).^336^ There are several mRNA vaccines against bacteria and parasites,^337^ but they are still under preclinical evaluation.^338^ Collectively, these studies indicated that mRNA vaccination is a promising strategy against infectious diseases, although further research and development are urgently required for some of these diseases, such as AIDS.

**Table 3:** Clinical trials of mRNA drugs

Therapeutics	Therapeutic strategy	Indication	Phase	Name	Encoded protein	Vector/route	Clinical result
Infection disease	Vaccine	COVID-19	III	mRNA-1273	S-2P	LNP/i.m.	>90% efficacy (lower efficacy for the delta variant of SARS-CoV-2)^536,553^
			III	BNT162b2	S-2P	LNP/i.m.	>90% efficacy (lower efficacy for the delta variant of SARS-CoV-2)^554,555^
			II/III	BNT162b1	Trimerized RBD	LNP/i.m.	Patients have developed 1.1–4.6 times GMT after two doses^556^
			III	ARCoV	RBD	LNP/i.m.	Not reported^276^
			II/III	mRNA-1273.211	Wuhan-Hu-1 and B.1.351-variant S-2P	LNP/i.m.	Not reported^557,558^
			II	ARCT-021	naïve S protein	LNP/i.m.	Not reported^293^
			I/II	MRT5500	S-2P with a furin cleavage-site mutant	LNP/i.m.	Not reported^559^
			I/II	ChulaCov19	Transmembrane S protein	LNP/i.m.	Not reported^560^
		Rabies	I	CV7201	Rabies virus glycoprotein	Protamine-condensed mRNA/i.d.	70.3% participants had WHO-specified protective antibody titers^330^
		Influenza	I	CV7202	RABV-G protein	LNP/i.m.	No result posts^351^
			I	VAL-506440	Hemagglutinin glycoprotein	LNP/i.d.	Vaccines elicited similar antibodies level with licensed vaccines^385^
		Acute HIV infection	I	AGS-004	Gag, Nef, Vpr and Rev	DCs electroporated with mRNA/i.d.	All six patients restarted antiretroviral therapy.^561^
	Antibody	Chikungunya virus Infection	I	mRNA-1944	anti-Chikungunya antibody	LNP/not reported	No reported
	Gene editing	acute HIV infection	I	SB-728mR-HSPC	ZFN targeting CCR5 gene	HSCT electroporated with mRNA/infusion	No reported^562^
			I/II	SB-728mR-T	ZFN targeting CCR5 gene	CD4^+^ T cell/infusion	HIV DNA decreased in most patients^485^
Cancer immunotherapies	Vaccine	Melanoma	I	Lipo-MERIT	TPTE, NY-ESO-1, MAGE-A3 and tyrosinase	Lipoplex/i.v.	1/3 PR, 1/3 SD, and 1/3 RSM^367^
			I	IVAC MUTANOME, RBL001/RBL002	Neoantigens	Nake mRNA/i.n.	5/13 progression and 8/13 PR^366^
			I/II	None	mRNA copy of tumor	Nake RNA/i.d.	9/15 death, 2/15 PR and 4/15 SD^360^
			I	None	p53, survivin, and hTERT	DCs/i.d.	9/22 SD and 13/22 PD^226^
			II	None	gp100 and tyrosinase	DCs/i.d. and i.v.	No improved clinical outcome combined with cisplatin^363^
			I	None	mTRP-2	DCs/i.d.	6/10 PR and 4/10 PD^227^
			I/II	None	mRNA from autologous tumor material	mRNA-electroporated DCs/i.n. or i.d.	3/31 SD and 25/31 PD^361^
			I/II	None	gp100 and tyrosinase	DCs/i.n. or i.d.	5/12 SD (B), 13/16 (A) and 7/12 (B) progression^365^
			I/II	None	gp100 and tyrosinase	DCs/i.n.	2/14 patients SD^364^
			I	TriMixDC‑MEL	MAGE- A3, MAGE-C2, tyrosinase and gp100	DCs/i.d.	71% of patients alive and free of disease^362^
		CRPC	I/II	CV9103	PSCA, PSMA PSA, and STEAP1	Protamine-condensed mRNA/i.d.	44 patients had 29.3 months median OS^384^
		NSCLC	I/II	CV9201	NY-ESO-1, 5T4, MAGE-C1, MAGE-C2, and survivin	Protamine-condensed mRNA/i.d.	9/29 SD and 20/29 progression^358^
			I/II	CV9202	NY-ESO-1, MAGE-C1, MAGE-C2, survivin, 5T4, and MUC1	Protamine-condensed mRNA/i.d.	12/26 progression^563^
		Renal cell cancer	I/II	None	Her2/neu, MAGE-A1, CEA, survivin, MUC1, Telomerase	nake RNA/i.d.	30 patients had 24.5 months median OS^373^
			II	AGS-003	Not reported	DCs/i.d.	30.2-month median OS with sunitinib^372^
		AML in CR with high relapse risk	II	None	WT1	DCs/i.d.	6/30 CR1 and 11/30 CR2^76^
			II	GRNVAC1	hTERT	DCs/i.d.	11/19 CR^74^
		Glioblastoma	I	None	pp65	DCs/i.d.	3/6 PR* and 3/6 progression^370^
		Glioma	I/II	None	mRNA copy of tumor	DCs/i.d.	5/7 progression^369^
	CAR-T	Bread cancer	I	cMet RNA CAR T cells	CAR specific for c-Met	T cell electroporated with mRNA/intratumoral injections	Well tolerance^473^
		Hodgkin lymphoma	I	None	Anti-CD19-CAR	T cell electroporated with mRNA/infusion	Well tolerance^564^
Genetic disorders	Protein replacement	Methylmalonic acidaemia	I/II	mRNA-3704	Methylmalonyl-CoA mutase	Unknown/i.v.	No reported
	Protein replacement	Propionic acidaemia	I/II	mRNA-3927	Propionyl-CoA carboxylase	Unknown/i.v.	No reported
	Protein replacement	OTD	I/II	MRT5201	Ornithine transcarbamylase	LNP/i.v.	No reported
	Protein replacement	Cystic fibrosis	I/II	MRT5005	CFTCR	Unknown/inhalation	No pattern of increases in ppFEV1
	Gene editing	TAP	I	NTLA-2001	CRISPR–Cas9	LNP/i.v.	Decreases in serum TTR protein^565^
Metabolic disorders	Protein replacement	Type II diabetes	I	AZD8601	VEGFA	Nake mRNA/i.d.	Transient skin blood flow ^566^ improvement
Cardiovascular disease	Protein replacement	Heart failure	II	AZD8601	VEGFA	Nake mRNA/epicardial injection	No reported^426^

*EUA* Emergency Use Authorization, *CMA* conditional marketing authorization, *LNP* lipid nanoparticle, *S-2P* full-length S protein with two proline mutations, *i.v.* intravenous injection; *i.n.* intranodal injection; *i.d.* intradermal injection, *PR* progression free, *SD* stable disease, *TR* tumor regression, *OS* overall survival, *RBD* receptor-binding domain of spike protein, *modified S protein* SARS-CoV-2 spike protein is locked in the prefusion conformation by two proline mutations, *GMT* geometric mean titers, *TriMix-mRNA* mRNAs encoding CD40 L, CD70 and caTLR4, *CRPC* castration-resistant prostate cancer, *NSCLC* non-small cell lung cancer, *AML* acute myeloid leukemia, *SCA* prostate stem cell antigen, *PSA* prostate-specific antigen, *PPSMA* prostate-specific membrane antigen, *STEAP1* six-transmembrane epithelial antigen of the prostate 1, *mTRP-2* murine tyrosinase-related peptide-2, *hTERT* human telomerase reverse transcriptase, *TAA* tumor-associated antigen, *ZFN* zinc finger nucleases, *HSCT* hematopoietic stem cell transplantation, *CAR* chimeric antigen receptor, *OTD* ornithine transcarbamylase deficiency, *TAP* transthyretin amyloidosis with polyneuropathy, *CFTCR* cystic fibrosis transmembrane conductance regulator, *ppFEV1* percent predicted forced expiratory volume in 1 second, *CRISPR-Cas9* a clustered regularly interspaced short palindromic repeats/Cas9 gene-editing system

##### Influenza virus mRNA vaccine

Nachbagauer et al. selected the conserved HA stalk domain, matrix-2 ion channel, nucleoprotein, and broadly reactive neuraminidase as antigens to provide universal protection against the influenza virus. The vaccines used LNP to deliver m1Ψ mRNA and protected mice from challenge with H1N1 virus at 500-fold the median lethal dose (intradermally, an ionizable cationic lipid/phosphatidylcholine/cholesterol/PEG-lipid (50:10:38.5:1.5 mol/mol)).^339^

##### HIV mRNA vaccine

Mariano Esteban used vaccinia virus Ankara vectors to load unmodified and 1-methyl-3′-pseudouridylyl modified mRNA (m1Ψ mRNA) encoding HIV-1 Gag, Pol and Nef proteins (an ionizable cationic lipid/phosphatidylcholine/cholesterol/PEG/lipid (50:10:38.5:1.5 mol/mol)).^340,341^

##### RSV mRNA vaccine

Respiratory syncytial virus mRNA vaccine mRNA-1777 showed safety and tolerability in a phase I clinical trial.^342^ Bett et al. used LNP to deliver mRNA encoding full-length wild-type F protein, a full-length mutated F protein, a truncated secreted trimeric form of F protein, a secreted prefusion-stabilized F protein, and the full-length wild-type and prefusion-stabilized forms evoked a higher immune response (LNP formulation: asymmetric ionizable amino lipid, DSPC, cholesterol, and poly(ethyleneglycol) 2000-dimyristoylglycerol (PEG2000-DMG) in a molar ratio of 58:30:10:2, respectively).^343^

##### HSV mRNA vaccine

Friedman et al. developed a trivalent mRNA vaccine targeting herpes simplex virus type 2 glycoproteins C, D, and E. Compared to a trivalent protein vaccine, a m1Ψ-modified mRNA vaccine provided better protection.^344,345^ Friedman et al. compared the HSV mRNA vaccine and protein vaccine that used the same antigens (glycoproteins C2, D2, E2), and the former induced a stronger immune response and memory.^346^

##### VZV mRNA vaccine

Vora et al. used LNP to deliver m1Ψ mRNA encoding varicella-zoster virus (VZV) gE antigen, which showed an effect comparable to that of a protein vaccine adjuvanted with AS01B (LNP formulation, ionizable lipid: DSPC:cholesterol:PEG-lipid, 50:10:38.5:1.5).^347^

##### Human cytomegalovirus (HCMV) vaccines mRNA vaccine

Permar et al. used LNP to deliver m1Ψ mRNA encoding full-length glycoprotein B protein that evoked a more durable immune response than the protein vaccine adjuvanted with MF59 (LNP formulation, an ionizable cationic lipid (proprietary to Acuitas), phosphatidylcholine, cholesterol, and PEG-lipid (50:10:38.5:1.5, mol/mol).^348^ Similarly, they developed HCMV vaccines using mRNA encoding glycoprotein B and the pentameric complex that induced significant immune responses in nonhuman primates with preexisting immunity against HCMV.^349^

##### Rabies virus mRNA vaccine

Rabies virus causes a zoonotic infection, imposing an estimated 59,000 deaths each year. Despite effective vaccines, rabies remains one of the most distressing diseases worldwide, owing to unobtainable treatment and complicated vaccine regimens (requiring 4 doses). CureVac AG developed a rabies virus (RABV) mRNA vaccine, CV7201, that uses the cationic protein protamine to encapsulate mRNA encoding the glycoprotein of rabies virus to treat rabies. CV7201 was temperature stable and successfully elicited a WHO-specified antibody response in 70.3% of participants via three i.d. vaccination (NCT02241135).^330^ Based on CV7201, CureVac AG optimized the LNP formulation and developed CV7202, which uses the same mRNA antigen as CV7201. The optimized LNP includes an ionizable amino lipid, a PEG-modified lipid, phospholipid, and cholesterol.^350^ CV7202 showed good tolerance in a clinical trial (NCT03713086).^351^ Luis-Alexander Rodriguez used a CNE to encapsulate saRNA encoding alphavirus RNA-dependent RNA polymerase and the rabies glycoprotein G.^352^

##### Dengue virus mRNA vaccine

Richner et al. used LNP to encapsulate mRNA encoding the envelope and membrane structural proteins of Dengue virus serotype 1.^353^

##### Other mRNA vaccines

Spiropoulou et al. used LNP to encapsulate mRNA encoding the soluble Hendra virus glycoprotein, which protected 70% of Syrian hamsters from lethal NiV challenge.^354^ Sigal et al. developed an mRNA vaccine against ectromelia virus using mRNA encoding EVM158.^355^

#### mRNA cancer vaccines

Immunotherapy has been an evolving and promising cancer treatment by stimulating the immune system, including immune checkpoint blockade (ICB), chimeric antigen receptor T cells (CAR-T cells), and vaccines.^356^ Unlike ICB releasing immunosuppression and CAR-T cells directly killing tumor cells, a cancer vaccine initiates and amplifies the antitumor immune response by APCs, especially DCs.^357^ mRNA cancer vaccine platforms have been developed and have achieved encouraging outcomes based on their unique efficacy in pushing the cancer immunity cycle and safety. mRNA vaccines for castration-resistant prostate cancer and non-small-cell lung cancer were clinically evaluated.^358^ Meanwhile, mRNA vaccines for melanoma, glioblastoma, AML, and renal cell carcinoma (RCC) demonstrated an active response to immunotherapy, which deserves intensive further exploration in the mRNA vaccine field.^359^

##### Melanoma

Three non-DC-based and seven DC-based mRNA vaccines have been tested clinically. Among them, one non-DC-based^360^ and one DC-based mRNA vaccine^361^ used complete mRNAs from tumor cells, and other vaccines selected TAAs and encoded them into mRNAs. Notably, all DC-based mRNA vaccines failed to significantly improve clinical outcome in metastatic melanoma patients, and more than half of the participants developed disease progression during clinical trials, and intranodal (i.n.) vaccination failed to improve the efficacy of DC-based mRNA vaccines and had a lower response rate than i.d. vaccination (NCT01278940).^226,361–365^ TriMix-mRNA (containing mRNAs coding immunostimulatory molecules: CD40 L, CD70, and caTLR4) was implemented to improve DC-based vaccine efficacy.^362,364^ In addition, the BioNTech company developed a personal mRNA vaccine for metastatic melanoma, had no detectable lesions on radiology, and remained recurrence-free after 23 months of i.n. vaccination (NCT02035956)^366^ and exploited LNP to generate an anti-melanoma mRNA vaccine, which attributed to regression of a suspected metastasis in an intravenously vaccinated patient (NCT02410733).^367^ Due to the inconsistent data, further research may help confirm that mRNA vaccines can serve as an immunotherapy for melanoma.

##### Glioblastoma

mRNA vaccination has been considered a promising strategy to treat glioblastoma.^368^ DC-based mRNA vaccines were generated by using mRNA copies of glioblastoma in patients and prolonged progression-free survival 2.9 times compared with matched controls (NCT00961844).^369^ Likewise, pre-conditioning the vaccine site with a potent recall antigen such as tetanus/diphtheria (Td) toxoid can significantly improve the efficacy of tumor-antigen-specific DCs, thus increasing DC migration bilaterally and significantly improving glioblastoma patients survival.^370^ A DC-based mRNA vaccine was developed to improve mRNA-pulsed DC homing to lymph organs (NCT00639639, relevant results have not yet been announced).

##### Acute myeloid leukemia

Two DC-based mRNA vaccines have been developed to reduce the relapse risk of AML patients with complete remission (NCT00510133 and NCT00965224). Electroporation DCs with WT1 mRNAs improved relapse-free survival in vaccination responders compared with nonresponders. Another study exploited mRNA encoding human telomerase reverse transcriptase, and i.d. vaccinations resulted in 11 of 19 patients in complete remission with a 52-month median follow-up.^74,371^ Notably, mRNA vaccines may be unsuitable for patients with processive AML because they depend on the immune system to exert function, while AML can impair patients’ immune system.^76^

##### Renal cell carcinoma (RCC)

RCC continues to have high mortality rates, and two mRNA vaccines have been developed to treat RCC. DC-based mRNA vaccines showed moderate efficacy (NCT00678119) for advanced RCC treatment.^372^ Another anti-RCC mRNA vaccine is directly administered to patients via the i.d. route, and the vaccine-specific immune response seems to be related to the long-term survival of RCC patients.^373^

#### Tolerance to mRNA cancer vaccines

Tumors boast many mechanisms to evade efficacy immunosurveillance by upregulating immunosuppressive molecules and corresponding cells under the antitumor pressure of immunotherapy, resulting in the induction of peripheral tolerance and central tolerance and significantly impairing immunotherapy efficacy.^374^ The treatment strategies of ICBs are widely exploited to break immune tolerance, including anti-PD-1 antibodies,^366^ anti-CTLA-4 antibodies,^375^ and PD-L1 siRNA.^376^ Unlike ICBs, natural killer (NK) cells may be favorable for overcoming the tolerance mechanism, which is related to NK cells eliminating tumor cells without the presentation of MHC I molecules.^366,377^ TAAs, as self-antigens, have central tolerance due to the clonal deletion of autoreactive lymph cells during ontogenesis.^378^ Neoepitopes can bypass central tolerance with high immunogenicity because they are never present in normal tissues and generate the accumulation of gene mutations in cancer cells (including driver mutations and passenger mutations).^379^ Therefore, neoepitopes were applied to overcome the central tolerance of cancer vaccines and address the issue of tumor heterogeneity. The personal mRNA vaccine has shown relatively favorable clinical efficacy, but some patients were unavailable for vaccination due to disease progression, and merely a portion of neoepitopes successfully induced a specific immune response in patients.^366^ Recently, several clinical trials have been launched to further evaluate the antitumor efficacy of personal mRNA vaccines (NCT03313778, NCT02316457, and NCT03468244, relevant results have not yet been announced).^380,381^ Collectively, based on the complexity of tumor pathogenesis, codelivery of multiple therapeutic mRNAs has great potential to defeat cancer.

#### The safety of mRNA vaccines

mRNA vaccines have sufficient safety with good tolerance, and their adverse events (AEs) are generally mild to moderate, including injection site reactions such as pain, swelling, erythema, and influenza-like illnesses such as fatigue, myalgia, pyrexia, and chills.^382,383^ In particular, the antirabies mRNA vaccine CV7201 caused unexpected grade 2 Bell’s palsy in a healthy adult with intramuscular (i.m.) vaccination,^330^ and CV9130 caused urinary retention in three patients with prostate cancer, while urinary retention is also a common symptom in prostate cancer.^384^ The CV9201 vaccination also caused a grade 3 asthma attack in 1 patient, abnormal thyroid-stimulating hormone in nine patients, and increased antinuclear antibody in five patients.^358^ DC vaccines seldom caused grade 3 AEs.^361^ The severity of AEs relates to the administration route and dosage.^352,385^ Notably, it seems that i.d. vaccination has a higher AE frequency than i.m. administration: CV7201 caused 7 of 10 grade 3 AEs in the i.d. groups (64 participants), only 3 AEs in the i.m. group (37 participants).^330^ mRNA vaccines are a practical platform to improve the safety of vaccines by changing antigen sequences and modifying protein structures. Antibody-dependent enhancement (ADE) is a phenomenon in which preexisting antibodies promote viral infection of host cells and lead to increased virulence.^386^ mRNA encoding an E protein mutation without a conserved fusion-loop epitope was employed to enhance the safety of the anti-Zika mRNA vaccine and avoid potential ADE risk.^387^ Furthermore, mRNA encoding the RBD instead of its parental protein reduced the harmful immune response induced by vaccines.^388^

#### Adjuvants for mRNA vaccines

Adjuvants are essential for mRNA-based therapeutics, especially mRNA vaccines, which can amplify and direct immune responses and modulate the magnitude and type of certain subsets of T helper, IgG subclasses, or mucosal antibody responses. There are a few adjuvants approved by the FDA for use in humans, including aluminum salts, MF59, AS01, AS03, AS04, and CpG 10181.^389^ For mRNA vaccines, the sources of adjuvants mainly include the following five categories: (1) the self-adjuvant efficacy of IVT mRNA; (2) the immune-activating protein encoded by the mRNA (e.g., CD70, CD40 L and TriMix-DC); (3) direct-acting adjuvants: pathogen-associated molecular patterns and danger-associated molecular patterns (e.g., TLRs, helicases, NODs, and inflammasome agonists); (4) mRNAs complexed with specific reagents (protamine, lipid reagent); and (5) adjuvants that can promote DC recruitment, proliferation, and cross-presentation, such as GM-CSF and Fms-like tyrosine kinase 3 ligand (FLT3 L).^390–392^ Exogenous mRNAs have an inherent immunostimulatory effect due to their recognition by a variety of innate immune receptors, which allow them to stimulate the innate immune response in favor of vaccination, but they induce mRNA degradation and inhibit antigen expression, which are detrimental to maintaining the activity of mRNA therapeutics.^78,393^ Previous research has indicated that nucleoside modifications improved mRNA translation efficiency (Ψ, 5mC, Ψ/5mC or N1-methyl-pseudouridine/5-methylcytidine), and the pseudouridine/5-methylcytidine (Ψ/5mC)-modified mRNA partly suppressed the innate immune activation by mRNA vaccines and increased the encoding protein levels (firefly luciferase) up to 100-fold in vitro and 20-fold in the spleen of mice.^394,395^ Paradoxically, studies also showed that Ψ modification increased the immune stimulation function of mRNAs and failed to enhance mRNA translation efficiency.^396,397^ This opposite conclusion may be related to variations in RNA sequence optimization, stringency of removal of dsRNA contaminants by mRNA purification, and the level of innate immune sensing in targeted cell types.^6^ Another efficacious adjuvant strategy is to encode immunomodulatory proteins used as adjuvants with mRNAs, such as TriMix, which encodes a combination of three immune-activating proteins: CD70, CD40 ligand (CD40 L), and constitutively active TLR4 (caTLR4).^398^ Numerous cancer vaccine studies have shown that TriMix mRNA is associated with the stimulation of DC maturation and the generation of potent cytotoxic T lymphocyte (CTL) responses.^399^ DCs electroporated with mRNA encoding the costimulatory molecule 4-1BB ligand (4-1BBL) and CD40 L enhanced the proliferation and function of HIV-specific CD8^+^ T cells and increased the secretion of cytokines.^400^ Other costimulatory molecules, including CD83 and tumor necrosis factor receptor superfamily member 4 (TNFRSF4; also known as OX40), can also be encoded by mRNA and electroporated DCs, resulting in a significant increase in the immunostimulatory activity of DCs.^401,402^ Recently, a novel mRNA vaccine against SARS-CoV-2 also incorporated the costimulatory molecule CD40 L as an adjuvant to activate professional APC.^403^ Pattern recognition receptor ligands act as adjuvants to induce innate immunity and target APCs, thereby influencing the adaptive immune response. Pam3, a lipopeptide adjuvant recognized by TLR1 and TLR2, was incorporated into LNP, which enhanced mRNA-mediated cancer immunotherapy by stimulating different TLR subclasses.^404^ Double-stranded RNA (dsRNA) that is produced during the replication of viruses can powerfully induce natural immunity. Poly (I: C), a synthetic analog of dsRNA, is considered to be a TLR3 agonist that induces the production of IL-12 and type I IFN, promotes antigen cross-presentation to MHC class II molecules, and improves the generation of cytotoxic T cells.^405^ However, nucleic acid adjuvants have certain restrictions related to instability and easy degradation after drug administration, so delivery systems are generally considered to optimize them. Recently, an anionic poly I:C-derived double-stranded RNA adjuvant was complexed with chitosan to synthesize polyplexes to stimulate DC maturation, promote antigen presentation, and initiate cytotoxic T cells, which showed certain therapeutic efficacy in cancer treatment.^406^ Monophosphorylate lipid A activates the immune system via TLR4 without affecting mRNA translation.^407^ Synthetic CpG oligodeoxynucleotides (ODNs) are TLR-9 agonists that can induce the production of type I IFN and proinflammatory cytokines and generate Th1-type cellular and humoral immune responses.^408,409^ The hepatitis B vaccine HBsAg-1018 (HEPLISAV-B™) containing CpG-ODN as an adjuvant has been approved by the US Food and Drug Administration for use in adults.^410^ RNAdjuvant^®^ (CureVac AG), an RNA-based TLR-7/8/RIG-I agonist consisting of a single-stranded, noncoding, cap-free RNA sequence containing multiple poly(U) repeat sequences, is a potent Th1-driven adjuvant that induces high levels of IFN-γ and has played a role in multiple tumor treatment studies.^411,412^

Other adjuvants that promote DC recruitment, proliferation, and cross-presentation, such as GM-CSF, were combined with naked mRNA to induce mainly a Th1 immune response, while naked mRNA alone induced a Th2 response.^413^ FLT3 L plays an important role in in situ vaccination, and the confounding protein FLT3 L also improves therapeutic immunity induced by naked mRNA.^414,415^ Overall, adjuvants reveal a critical role in mRNA-encoding antigens expression and initiating durable protective immunity, and have huge application prospects in mRNA-based therapeutics.

#### mRNA-based protein replacement therapies

Protein replacement treatment has an extensive application in replacing missing or defective proteins with favorable proteins.^50^ mRNA-based therapeutics have become a new pillar for protein replacement therapy, which has been extensively explored in various fields, including cardiac diseases,^416^ lung diseases,^417^ hematologic diseases,^418^ metabolic diseases,^419^ cancer,^420^ orthopedic diseases,^421^ neurogenic disorders,^422,423^ muscle atrophy, and so on.^50,424^ However, the majority of mRNA-based therapies for protein replacement are in the preclinical status, and only mRNA drugs encoding vascular endothelial growth factor (VEGF, NCT03370887) and CFTR (NCT03375047) have entered clinical development. To date, the most extensive efforts have been made in protein replacement therapeutics for cardiac diseases, focusing on heart failure and myocardial infarction.^416^ VEGFA mRNA treatment (AZD8601) protected mice from heart failure and significantly reduced apoptosis of myocardial cells with increased capillary density,^425^ and corresponding efficacy evaluation is ongoing in clinical trials (NCT03370887).^426^ However, testing an mRNA-based therapeutic also encouraged its application in protein replacement therapies for various lung diseases, especially genetic lung diseases.^417^

##### Cystic fibrosis

Cystic fibrosis is a life-limiting autosomal-recessive disease caused by mutations in the CFTR gene, while CFTR-mRNA transfection markedly restores impaired CFTR function in vitro.^427^ Nasally administered LNPs-CFTR mRNA was reported to result in recovery of up to 55% of the net chloride efflux characteristic in healthy mice.^428^ Furthermore, MRT5005, as an mRNA-based CFTR protein, has entered phase I/II clinical research.^148^

##### Hematologic diseases

Preclinical studies on mRNA-based protein replacement therapy have tested hematologic diseases.^429^ Hemophilia is a group of bleeding disorders for blood coagulation factor deficiency, including hemophilia A (factor VIII deficiency) and hemophilia B (factor IX deficiency).^430^ mRNA-based protein replacement can correct hematologic disorders by delivering corresponding factors in the template for mRNA. LNPs encapsulated mRNAs encoding different FVIII variants (F8 LNP) had rapid induction and durable FVIII expression in hemophilia A mice.^431^ FIX mRNA was delivered to FIX-knockout mice by using a series of lipidoids named TTs (corresponding lipid-like nanoparticles named TT-LLNs), which restored FIX function in FIX-knockout mice.^432^ Termed lipid-enabled LUNAR LNPs encapsulating hFIX mRNA were developed to treat hemophilia B mice, contributing to a rapid pulse of FIX within 4–6 h and a stable duration for up to 4–6 days.^433^

##### Metabolic diseases

The application of mRNAs also represents a promising solution for metabolic diseases that currently lack efficacious treatments, such as hepatorenal tyrosinemia, acute intermittent porphyria, Fabry disease, glycogen storage disease type 1 A, Crigler-Najjar syndrome type 1, and ornithine transcarboxylase deficiency.^418,419^ Hepatorenal tyrosinemia is a rare genetic metabolic disease caused by tyrosine degradation disorder due to a fumarylacetoacetate-hydrolase mutation, which can result in multiple organ damage.^434^ Cheng et al. designed and optimized 5A2-SC8 mRNA-loaded dendrimer LNPs to carry fumarylacetoacetate-hydrolase mRNA, which rendered FAH knockout mice statistically significant for liver function, similar to wild-type C57BL/6 mice.^166^ Acute intermittent porphyria is caused by the haploinsufficiency of porphobilinogen deaminase, which induces neurovisceral attacks associated with increased hepatic heme demand.^435^ LNP-encapsulated mRNA was used to induce dose-dependent expression of human porphobilinogen deaminase in mouse hepatocytes.^435^ This replacement therapy rapidly normalized urine porphyrin precursor excretion and counteracted porphyria attack in deficient mice, rabbits, and nonhuman primates. Methylmalonic acidemia, a genetic metabolic disease primarily caused by the loss of methylmalonyl-CoA mutase activity, results in approximately 20% mortality.^436^ LNP-encapsulated mRNA was delivered to systemically express functional mitochondrial methylmalonyl-CoA mutase in methylmalonic acidemia mice with a reduction of 75%-85% in plasma methylmalonic acid.^437^ A hybrid mRNA technology delivery system was exploited to load ornithine transcarboxylase mRNA, which restored the levels of plasma ammonia and urinary orotic acid and prolonged the survival of relatively deficient mice.^438^ Fabry disease is a lysosomal storage disorder caused by the deficiency of α-galactosidase A, resulting in cardiomyopathy and end-stage renal disease. Fabry disease can be improved by using nanoparticles sustainably to deliver α-galactosidase A mRNA into a mouse and nonhuman primate.^439^ Mutation of the SERPINA1 gene leads to alpha 1-antitrypsin (AAT) deficiency and damages the liver where the AAT protein is produced. Karadagi et al. identified mRNA encoding human AAT in primary human hepatocytes and developed it into LNP formulations. An in vivo study showed that secreted AAT protein increased from 1.14 to 3.43 µg/mL in media from primary human hepatocytes.^440^ mRNA-based protein replacement also provides an alternative to tumor treatment. Phosphatase and tensin homologue deleted on chromosome 10 (PTEN) is a potent tumor suppressor gene that is missing or mutated in many human cancers. PTEN inhibited the PI3K-AKT pathway and enhanced apoptosis of prostate cancer cells.^441^ Polymer–lipid hybrid nanoparticles were employed to systemically deliver PTEN mRNA and significantly inhibited the growth of disseminated metastatic and intratibial orthotopic prostate cancer in PTEN-null mice.^442^ Similarly, polymer–lipid hybrid nanoparticles were modified with the redox-responsive polymer PDSA and applied to transmit p53 mRNA (another gene encoding a tumor suppressor), and the results showed that the p53 mRNA NPs arrested the cell cycle and induced apoptosis, contributing to significant growth inhibition of p53-null HCCs and NSCLCs and improving the sensitivity of tumor cells to rapamycin inhibitors.^167^ In addition, mRNA encoding an anti-angiogenic protein, soluble fms-like tyrosine kinase 1,^443^ also efficiently inhibited pancreatic tumors; the liposome-protamine-IL-22BP mRNA complex strongly inhibited C26 tumor growth in both a peritoneal metastasis model and subcutaneous xenograft model.^444^

#### mRNA encoding a peptide/protein

The function of a peptide/protein encoded by mRNA is the key factor in the selection of therapeutics targeting cells, which directly influences mRNA therapeutic design.^445^ Precise delivery is required to target cells with appropriate protein convertase or endoprotease for the peptide that needs posttranslational modification to assemble them into functional types.^446^ Proteins need to be secreted outside of the cells to exert their function. Thus, mRNAs need to be conveyed to cells with natural secretion functions; otherwise, it is necessary to insert the mRNA sequence of the corresponding signal peptide near the ORF of the secretory protein.^447^ Encoded peptide/protein antigens can also give rise to a heterogeneous immune response even if they are involved in the same vaccine.^448^ A trivalent vaccine using three mRNAs was generated to encode different proteins, while these three antigens contributed to different IgG levels.^94^ Similarly, Sahin et al. designed neopeptide-encoded mRNAs, while the magnitude of the immune response varied from peptide to peptide, which indicates that the mRNA vaccine can be improved by selecting strongly responsive antigens; however, the underlying mechanism is far from fully clear, and it is difficult to ensure that encoded peptides/proteins all possess high immunogenicity.^366^ Moreover, the encoded peptide/protein greatly impacts its sustained expression. Holtkamp et al. observed that a fluorescent protein sustained high-level expression up to 120 h in mRNA format, while expression duration was dramatically reduced using immunodominant peptide from OVA in a similar mRNA format.^46,379^ Notably, the duration of protein expression plays a role in mRNA therapeutic efficacy. For example, migratory DCs in the skin need to spend 48 h trafficking to the T cell zoo and another couple of hours evoking a de novo CD8^+^ T cell response after delivery of mRNA into DCs.^449–451^ Therefore, mRNA encoding a peptide/protein theoretically needs to be present on the surface of migratory DCs for at least 48 h for mRNA vaccines with subcutaneous or i.d. administration. Note that in most cancer mRNA vaccines, there is a sharp drop in peptide/protein expression at approximately 24 h after DC transfection or vaccine immunization.^46,452,453^ However, it remains unclear whether a longer duration is needed, which requires further research on the relationship between the kinetics of peptide/protein expression and mRNA vaccine (or therapeutic) efficacy.^454^

#### mRNA-based gene editing therapeutics

Gene editing has a torn pace of application in various fields driven by the rapid development of programmable nucleases,^423,424^ especially for cancer, infectious diseases, primary defects of the immune system, muscular dystrophy, and hematological disease.^455^ mRNA is widely used to deliver programmable nucleases.^456^ The three most important programmable nucleases, zinc finger nucleases (ZFNs),^457^ transcription activator-like effector nucleases (TALENs),^458,459^ and the clustered regularly interspaced short palindromic repeat (CRISPR)-associated protein (CRISPR/Cas) nuclease system,^460^ have all achieved efficient transfection and manipulated insertions/deletions mutations in the form of mRNA. mRNA is an attractive approach in gene editing therapy due to its transient expression without mutant risk, and currently, several clinical trials based on mRNA genetic editing are in progress.^461^ Here, we discuss the application of mRNA-based gene editing, as well as its future prospects and challenges.

##### CRISPR/Cas nuclease system

The advance of artificial endonucleases renders high-speed development of mRNA-based gene editing. mRNA drugs modulate cellular genomic information by encoding artificial endonucleases, such as ZFNs, TALENs, and more recently CRISPR/Cas nuclease systems.^462^ Generally, the three mRNA-encoded endonucleases were designed to achieve insertions/deletions (indels) and mutations by introducing a targeting DNA double-stranded break, followed by DNA repair through nonhomologous end joining or homology-directed repair pathways.^463^ CRISPR/Cas9 systems are currently the most frequently used gene-editing technology because of their convenience for design and implementation among three gene-editing tools.

##### mRNA-based T lymphocyte therapeutics

T lymphocytes are an intriguing target for their tremendous potential against cancer and infectious diseases, and electroporation is the main way to transform endonuclease-encoding mRNA into T cells in vitro.^464–466^ The main consideration is about the efficiency, specificity, and safety of engineering T lymphocytes via mRNA transfection, chemically modified sgRNAs and Cas9 mRNAs increased genome editing efficiency via electroporation into human primary T cells in vitro.^467^ Moreover, the delivery of Cas9 mRNA improved genome editing and reduced toxicity compared with DNA-based editing.^468^ In addition, TALEN endonuclease achieved high specificity and efficient genome editing in primary T cells. TALEN mRNA was electroporated into primary T cells and contributed to more than 50% CCR5 (HIV coreceptor) knockout with low off-target activity.^459^ Furthermore, the TCR knockout rate reached up to 81% in primary T cells after electroporation with TALEN mRNA and five guide RNAs from the CRISPR/cas9 system.^469^

##### mRNA-based autologous T cell therapeutics

Engineering T lymphocytes by mRNA electroporation ex vivo provides an efficient platform for the treatment of both viral infections and cancers without safety concerns associated with viral carriers.^470^ Generally, T cells acquire the ability to recognize tumor antigens via transgenic expression of a CAR or a high-affinity T cell receptor and subsequently exert therapeutic efficacy post infusion.^471^ Adoptive transfer of autologous T cells is a promising cancer immunotherapy but requires a high quantity and quality of autologous T cells, such as CAR-T cells.^472^ Nevertheless, genetic modification is a powerful approach to address these issues. Third-party donor T cells were electroporated with TCRa constant (TRAC) TALEN mRNA to develop large-scale manufacturing of T cells. Moreover, researchers disrupted the TRAC gene to avoid graft-versus-host reactions.^458^ To further improve the efficacy of CAR T cells, alemtuzumab, a chemotherapeutic agent, was administered to downregulate CD52 genes and synergistically promote engraftment by mediating lymphodepletion and immunosuppression, and it endowed TCR/CD52-deficient CD19 CAR T cells (dKO-CART19) with potent antitumor activity in an orthotopic CD19^+^ lymphoma murine model.^458^ Recently, the CRISPR/Cas system has emerged as a potential genome engineering tool for CAR T cell therapy. CAR and CRISPR were delivered by using lentiviral-loaded and electroporated mRNA, respectively, to engineer CAR T cells with HLA class I molecule, PD1 and TCR deficiency, and the CRISPR/Cas9 mRNA-disrupted allogeneic CAR T cells showed both efficient antitumor activity in vitro and in vivo.^473^ A hybrid ΔU3-sgRNA was designed and incorporated into the ΔU3-3′-long terminal repeat of a self-inactivating lentiviral vector, resulting in targeted TRAC locus cleavage and enrichment of highly homogeneous (>96%) CAR^+^ (>99%) TCR- populations and potent antileukemic activity of TCR-depleted CAR19 T cells in a human: murine chimeric tumor model.^474^ Together, CRISPR/Cas9 systems overcome allo-recognition and provide an alternative strategy to autologous T cells. Successful genome engineering was achieved by electroporation of mRNA coding for a CD19-CAR, with 94% CAR expression in > 80% viable T cells.^475,476^ The CTLs electroporated with mRNA encoding a CAR against CD19 exhibited significant CD19-specific antitumor activity after tail vein injection.^477^ Multiple infusions of CD19-directed RNA CAR T cells resulted in improved survival and sustained antitumor responses in a robust leukemia xenograft model preceded by lymphodepleting chemotherapy.^478^ In contrast to gene editing, Zhao and colleagues electroporated autologous T cells with mRNA encoding a CAR against mesothelin overexpression in pancreatic cancer, ovarian cancer, and mesothelioma.

Robust antitumor efficacy was demonstrated in a human disseminated mesothelioma xenograft model with multiple injections.^479^ However, inefficient trafficking to tumors has hindered ex vivo mRNA-based T cell treatment in clinical trials.^480^ Research has shown that T cell migration is improved by transfecting tumor-infiltrating T cells with mRNA encoding the chemokine receptor CXCR2.^481^ Recently, further clinical application of mRNA electroporated CAR-T cells was promoted by establishing clinical-scale production, and the mRNA encoding chondroitin sulfate proteoglycan to treat melanoma patients is under full GMP compliance, suggesting a potential value of the further clinical application.^482^ Currently, several studies of mRNA-based engineered CAR T cells have entered clinical safety and efficacy evaluations (NCT01837602, NCT02624258 and NCT03060356).^473^ Nonviral vectors have recently been designed for ex vivo mRNA delivery to human T cells considering the electroporation cytotoxicity. Olden et al. explored a series of cationic pHEMA-g-pDMAEMA polymers to deliver mRNA to CD4^+^ and CD8^+^ primary human T cells in vitro, which resulted in 25% transfection efficiency with high cell viability.^483^ Library screening approaches have been utilized to develop lipid/polymer-based mRNA delivery systems and provide a quick and easy method to recognize potential mRNA delivery systems for both preclinical and clinical engineering T lymphocytes. Billingsley et al. synthesized a library of 24 ionizable lipids and formulated them into LNPs, whose top-performing LNP renders CAR mRNA expression comparable to electroporation.^161^ McKinlay et al. generated a library of oligonucleotide transporters containing various lipid domains, which facilitated efficient mRNA release using amphiphilic CARTs and achieved a ninefold mRNA translation enhancement (80%) in lymphocytes in vitro compared to Lipofectamine 2000.^484^

##### mRNA-based CD4^+^ T cell therapeutics

To date, there is only one completed phase I study of CD4^+^ T cells modified at the CCR5 gene by ZFN mRNA in HIV-infected patients (NCT02388594).^485^ Challenges remain in cytotoxic gene delivery of the viral or electroporation methods, complex and expensive manipulations, and off-target efficacy of the gene-editing system. Encouragingly, very strong efforts have been made to explore nonviral and in vivo mRNA delivery for efficient and safe gene editing, which is worth looking forwards to in the future.^485^

##### mRNA-based stem cell therapeutics

mRNA-based genome editing has also been successfully applied to stem cells for many disease treatments.^486^ Previously, ZFN protein, mRNA, and DNA were delivered to a human cell line and mouse embryonic stem cells via a retrovirus vector and disrupted the targeted gene at frequencies of 15%, 15%, and >50%, respectively, indicating the universality of retroviral vectors.^487^ Kohn et al. further examined the efficiency, specificity, and mutational signatures of ZFN mRNA, TALEN mRNA, and CRISPR/Cas9 mRNA, which were electroporated into primary human hematopoietic stem and progenitor cells, and analyses revealed that ZFN mRNA has higher specificity than the other two endonucleases mRNA.^488^ ZFN mRNA enabled CD34^+^ to engraft in NOD-PrkdcSCID-IL2Rγ null mice with reserved multilineage potential compared with TALEN mRNA editing.^488^ For plasmid gRNA and Cas9 mRNA, their codelivery showed similar acute cytotoxicity with separate plasmid delivery, highlighting the need for further optimization of CRISPR/Cas9 delivery in primary human hematopoietic stem cells.^489^ Genome-editing approaches that innovatively transfect hematopoietic stem and progenitor cells with macaque-specific CCR5 ZFN mRNA ex vivo first modified multilineage and long-term repopulating cells in a large animal model and resulted in persistent in vivo tracking of genome-edited hematopoietic stem cells in a mutation-specific manner.^490^ Strategies for the transfection of stem cells are worth investigating for the ex vivo and in vivo delivery of endonuclease mRNA to facilitate clinical applications.

Ex vivo delivery of mRNA to stem cells has been explored for various purposes. Electroporation was used to transfer mRNA encoding EGFP into mesenchymal stem cells and H9 human embryonic stem (H9 hES) cells, both of which achieved 90% transgene efficiency.^491,492^ To provide a great alternative to pDNA, cationic carriers were explored to deliver mRNA encoding CXCR4 into mesenchymal stem cells and resulted in 80% positive expression rates of the target protein.^493^ In addition, numerous researchers have focused on improving the efficiency of mRNA transfection of stem cells. In vitro mRNA transcription was performed to characterize histone variant distribution in human embryonic stem cells.^494^ Researchers have successfully transdifferentiated insulin-producing cells to treat diabetes by using in vitro duodenal transcription factor 1 mRNA to transform the mouse pancreas into mesenchymal stem cells.^432^ Recently, HIV-1 Tat mRNA was delivered into bone marrow mesenchymal stem cells (BMSCs), confirming the inhibitory effect of HIV-1 Tat protein on the hematopoietic support function of hBMSCs.^495^

##### mRNA-based pluripotent stem (iPS) therapeutics

Genome editing of induced pluripotent stem (iPS) cells holds great promise in cell therapy and disease modeling.^496,497^ Many efforts have been made for genome editing of iPSCs using the CRISPR/Cas9 system.^498–500^ Transient delivery of Cas9 mRNA or protein is preferable for iPS clinical applications without mutation risk. Delivery of Cas9 in the form of mRNA has several advantages over direct protein delivery, including considerable protein molecule production from a single mRNA molecule and versatile mRNA engineering. A workflow capitalizing on the transient delivery of CRISPR/Cas9 mRNAs was presented to support the high-throughput development of gene-edited iPSCs. Subsequently, iPSCs can be differentiated into representative specific cell types of embryonic lineages for further research or potential clinical application. In addition, it was also applied to other gene-editing tools, such as ZFN mRNA and TALEN mRNA.^501^ However, RNA instability and off-target efficacy are challenging for clinical application.^502^ Hence, future efforts will pay attention to safe and efficacious delivery strategies of mRNA for further therapeutic purposes.

#### Combination therapeutics based on mRNA drugs

Recently, combined therapeutics have emerged as a powerful modality to treat malignancy, contributing to synergetic efficacy.^503^ ICB,^504^ CAR T cells ^265^ and cancer vaccines are three important immunotherapies for cancer treatment. ICBs can release the brake of T cell activation and function,^504^ but durable clinical benefit is only achieved in a minority of patients.^505^ The combination of ICBs and cancer vaccines has attracted considerable attention.^506^ The cancer vaccine can expand ICB efficacy by evoking a tumor-specific CD8^+^ T cell response to treat patients who lack preexisting CTLs and respond to ICBs^507,508^ and improve mRNA cancer vaccine efficacy.^366,509,510^ Recently, mRNA vaccines were amplified by CAR-T cells over 2 orders of magnitude by mimicking the dynamics of the secondary response following the initial reaction of T cells, which significantly increased median survival and contributed to the complete rejection of solid tumors in 6 of 10 mice compared to a single administration of CAR T cells.^511^ Apparently, mRNA-based therapeutics mainly focused on tumor immunotherapy and infectious disease, exploration of its potential and mechanism in other diseases is the next priority. Undoubtedly, mRNA-based therapeutics have become powerful and versatile tools to combat diseases.

## Conclusion and prospects

mRNA-based therapeutics have made great strides, achieving remarkable improvement in mRNA stability, function, and production during the past 30 years.^2^ mRNA drugs exploit cells as factories for antigen or functional protein production with promising efficacy and sufficient safety.^512^ Currently, a great deal of research focuses on varied applications of mRNA therapeutics, and a series of clinical trials are ongoing. mRNA vaccines have drawn considerable attention due to the important role of mRNA vaccines in controlling the SARS-CoV-2 pandemic.^513^ For vaccines against infection, the humoral immune response plays an important role in mRNA vaccine efficacy, especially IgG magnitude.^514^ The mRNA vaccine completely protected mice from influenza virus challenges with undetectable hemagglutination inhibition titers.^94^ Notably, mucosal immunity has also contributed significantly to defending against infectious diseases because many infections start from mucous membranes.^513,515,516^ Patel et al. systematically reviewed clinical trials of rotavirus vaccines following PRISMA guidelines, which displayed a consistent relationship between serum IgA and vaccine protection.^517^ Meanwhile, mucosal immunity may provide a wider protection than humoral immunity. The influenza virus vaccine with a higher nasal IgA level provided stronger protection than a lower IgA response, although the two vaccines had a similar serum IgG magnitude,^518^ and Tamura et al. also observed superior cross-reactivity of nasal IgA against heterologous influenza viruses compared to IgG.^519^ Moreover, mucosa immunity may play an important role in preventing the transmission of infection, and serum IgG possibly tends to prevent severe infectious diseases but no disease transmission.^515,520,521^ It is vital for vaccines to prevent COVID-19 transmission caused by asymptomatic carriers to counteract the current pandemic, which has demonstrated huge success. Currently, an intranasal vaccine was developed by regulating mucosal immunity against SARS-CoV-2, while the role of mucosal immunity is unclear in the prevention of SARS-CoV-2 transmission, which warrants further research to reveal the relationship between mucosal immunity and mRNA vaccines.^276^ Intriguingly, many mRNA vaccines tend to induce a Th-1-biased immune response through interferon signals, which may be related to mRNA delivery into the cytoplasm and translate antigen proteins that are largely processed on MHC I molecules and specifically activate the CD8^+^ T cell response.^522^ Together, mRNA vaccines have shown potent efficacy in defending against infectious diseases by humoral immune mucosal immunity, but cellular immunity needs to be assessed in detail in the future.

In recent decades, especially the last few years, we have witnessed great scientific advances in mRNA-based therapeutics. Current clinical efforts encompassing mRNA-based drugs are directed toward infectious disease vaccines, cancer immunotherapies, therapeutic protein replacement therapies, and genetic disease treatment. Opportunities and challenges in mRNA-based therapeutics coexist, and there are a large number of questions requiring clarification. (1) How can mRNA macromolecules be better delivered? (2) How can its inherent instability and degradation be improved by structure-based antigen design and delivery system-optimization? (3) How can its activation of the immune system be regulated? In essence, the clinical translation of mRNA-based therapeutics requires delivery technologies that can ensure stabilization of mRNA under physiological conditions. Improving the optimization technology of mRNA structure and engineering precision nanoparticles for mRNA-based therapeutics are also crucial points for the development of mRNA drugs as powerful and versatile tools to combat diseases. Built on the highly fueled interest and potential, we have full confidence to predict an accelerated pace in mRNA therapy studies and development in the next decade, possibly providing many solutions for the prevention and treatment of currently incurable diseases.
